# MAEST: accurately spatial domain detection in spatial transcriptomics with graph masked autoencoder

**DOI:** 10.1093/bib/bbaf086

**Published:** 2025-03-07

**Authors:** Pengfei Zhu, Han Shu, Yongtian Wang, Xiaofeng Wang, Yuan Zhao, Jialu Hu, Jiajie Peng, Xuequn Shang, Zhen Tian, Jing Chen, Tao Wang

**Affiliations:** School of Computer Science, Northwestern Polytechnical University, 1 Dongxiang Road, Xi’an 710072, China; Key Laboratory of Big Data Storage and Management, Ministry of Industry and Information Technology, Northwestern Polytechnical University, 1 Dongxiang Road, Xi’an 710072, China; School of Computer Science, Northwestern Polytechnical University, 1 Dongxiang Road, Xi’an 710072, China; Key Laboratory of Big Data Storage and Management, Ministry of Industry and Information Technology, Northwestern Polytechnical University, 1 Dongxiang Road, Xi’an 710072, China; School of Computer Science, Northwestern Polytechnical University, 1 Dongxiang Road, Xi’an 710072, China; Key Laboratory of Big Data Storage and Management, Ministry of Industry and Information Technology, Northwestern Polytechnical University, 1 Dongxiang Road, Xi’an 710072, China; General Surgery Department, The Affiliated Hospital of Northwest University: Xi’an No 3 Hospital, Xi’an 710018, China; School of Computer Science, Northwestern Polytechnical University, 1 Dongxiang Road, Xi’an 710072, China; Key Laboratory of Big Data Storage and Management, Ministry of Industry and Information Technology, Northwestern Polytechnical University, 1 Dongxiang Road, Xi’an 710072, China; School of Computer Science, Northwestern Polytechnical University, 1 Dongxiang Road, Xi’an 710072, China; Key Laboratory of Big Data Storage and Management, Ministry of Industry and Information Technology, Northwestern Polytechnical University, 1 Dongxiang Road, Xi’an 710072, China; School of Computer Science, Northwestern Polytechnical University, 1 Dongxiang Road, Xi’an 710072, China; Key Laboratory of Big Data Storage and Management, Ministry of Industry and Information Technology, Northwestern Polytechnical University, 1 Dongxiang Road, Xi’an 710072, China; School of Computer Science, Northwestern Polytechnical University, 1 Dongxiang Road, Xi’an 710072, China; Key Laboratory of Big Data Storage and Management, Ministry of Industry and Information Technology, Northwestern Polytechnical University, 1 Dongxiang Road, Xi’an 710072, China; School of Computer Science and Artificial Intelligence, Zhengzhou University, No. 100 Science Avenue, Zhengzhou 450001, China; School of Computer Science and Engineering, Xi’an University of Technology, No. 5 South Jinhua Road, Xi’an 710048, China; School of Computer Science, Northwestern Polytechnical University, 1 Dongxiang Road, Xi’an 710072, China; Key Laboratory of Big Data Storage and Management, Ministry of Industry and Information Technology, Northwestern Polytechnical University, 1 Dongxiang Road, Xi’an 710072, China

**Keywords:** spatial transcriptomics, spatial domain identification, joint domain detection, graph masked autoencoder, graph contrastive learning

## Abstract

Spatial transcriptomics (ST) technology provides gene expression profiles with spatial context, offering critical insights into cellular interactions and tissue architecture. A core task in ST is spatial domain identification, which involves detecting coherent regions with similar spatial expression patterns. However, existing methods often fail to fully exploit spatial information, leading to limited representational capacity and suboptimal clustering accuracy. Here, we introduce MAEST, a novel graph neural network model designed to address these limitations in ST data. MAEST leverages graph masked autoencoders to denoise and refine representations while incorporating graph contrastive learning to prevent feature collapse and enhance model robustness. By integrating one-hop and multi-hop representations, MAEST effectively captures both local and global spatial relationships, improving clustering precision. Extensive experiments across diverse datasets, including the human brain, mouse hippocampus, olfactory bulb, brain, and embryo, demonstrate that MAEST outperforms seven state-of-the-art methods in spatial domain identification. Furthermore, MAEST showcases its ability to integrate multi-slice data, identifying joint domains across horizontal tissue sections with high accuracy. These results highlight MAEST’s versatility and effectiveness in unraveling the spatial organization of complex tissues. The source code of MAEST can be obtained at https://github.com/clearlove2333/MAEST.

## Introduction

The complex tissue functions of multicellular organisms are fundamentally linked to the spatial organization of different cell types [1, 2]. Spatial transcriptomics (ST) technology enables researchers to map the spatial distribution of the entire transcriptome within histological tissue sections, significantly advancing our understanding of tissue structures [3, 4] and disease microenvironments [5, 6]. Recent advances in ST, including techniques like 10X Visium [7], Slide-seq [8], Stereo-seq [4], seqFISH [9], and MERFISH [10], enable the acquisition of spatially resolved gene expression data, providing powerful tools for studying the spatial patterns of gene expression in complex tissues.

Spatial domain identification, a key downstream task in ST, involves detecting coherent regions with similar spatial expression patterns. Current approaches for spatial domain identification typically rely on unsupervised clustering methods to assign capture sites (spots) to spatial domains. Traditional methods, such as K-Means [11] and Louvain method [12], focus solely on gene expression data to cluster spots into distinct domains. Seurat [13] generates clustering results by constructing a refined weighted K-nearest neighbor graph in PCA space, followed by a community detection algorithm to identify clusters. However, these approaches often yield discontinuous results, as they do not account for spatial information, which is crucial for identifying co-localized cells that may belong to the same domain. To address this limitation, BayesSpace [14] applies a Bayesian approach incorporating spatial neighborhood information to enhance resolution and improve clustering, ensuring adjacent spots with similar gene expression are grouped for more coherent spatial domains.

Graph neural networks (GNNs), with their ability to integrate gene expression and spatial information, provide a powerful approach for spatial domain identification in ST data [15, 16]. By generating expressive representations that capture both molecular and spatial context, GNNs enhance the accuracy of clustering and domain identification tasks. For example, SpaGCN [17] employs a graph convolutional network to identify spatial domains by integrating gene expression, spatial location, and histological images. DeepST [18] utilizes a GNN autoencoder paired with a denoising autoencoder to enhance latent representations, allowing data integration from multiple batches and technologies for more accurate spatial domain identification. STAGATE [19] adopts a graph attention autoencoder framework to integrate spatial information and gene expression profiles, enhancing model stability by sharing parameters between the encoder and decoder, thereby improving spatial domain identification accuracy. CCST [20] uses a deep graph infomax model [21], leveraging contrastive learning to optimize model parameters to improve performance in spatial domain identification. GraphST [22] integrates gene expression and spatial data using a graph convolutional network combined with graph contrastive learning and feature reconstruction, improving representation, model robustness, and spatial domain identification performance. For more detailed information about the models, please refer to Supplementary Table S1.

Although current spatial-aware domain detection methods have achieved superior performance compared to spatial non-aware methods, they often struggle with handling high dropout rates and effectively integrating spatial information with gene expression data, which can result in reduced accuracy and robustness in certain applications. In this work, we introduce a novel GNN model, MAEST, specifically designed for spatial transcriptomic data characterized by high dropout rates. This model fully integrates both local (neighborhood) and long-range node information, thereby improving the accuracy of spatial domain identification. MAEST employs a graph masked autoencoder to learn latent representations, enabling both denoising and signal enhancement. Additionally, MAEST incorporates graph contrastive learning, which prevents feature collapse by encouraging diverse and informative representations through contrasting positive (similar) and negative (dissimilar) samples. During the clustering stage, MAEST combines both one-hop and multi-hop spot representations, leveraging local and long-range spatial information to improve clustering accuracy. We extensively evaluated MAEST across multiple datasets, including the human brain, mouse hippocampus, olfactory bulb, brain, and embryo. The results demonstrate that MAEST accurately identifies spatial domains across diverse tissue types and effectively integrates multiple sections, outperforming current state-of-the-art methods in spatial domain identification.

## Materials and methods

**Figure 1:**
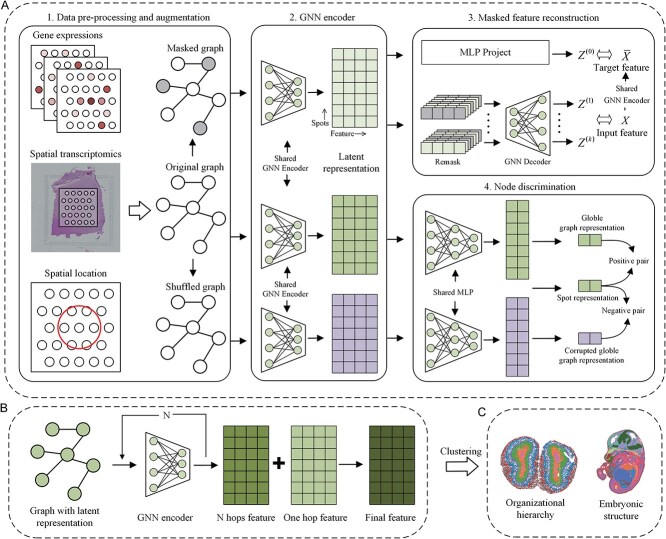
Overview of MAEST. (A) MAEST takes as input preprocessed gene expression data and a neighborhood graph constructed from spot coordinates, applying data augmentation for different tasks. Both the original and augmented graphs (masked and shuffled graphs) are passed through a shared GNN encoder for representation learning. The latent representation from the masked graph is used for the masked feature reconstruction task, while the representation from the shuffled graph supports the node discrimination task, contributing to model optimization. (B) The one-hop representation and multi-hop representation of the nodes are fused to obtain the final features. (C) The final features obtained through MAEST are used for spatial clustering.

In this study, we present MAEST, a deep learning framework leveraging GNNs to enhance the expressiveness of ST data and improve spatial domain recognition. The model’s training strategy consists of two core modules, as shown in Fig. 1A(3,4). The first, the masked feature reconstruction module, utilizes a GNN autoencoder to predict features of randomly masked nodes in the graph. This approach reduces data redundancy and prevents model collapse, resulting in more expressive node representations. The second, the node discrimination module, applies contrastive learning to classify nodes in the original graph versus those in a negative graph created through data augmentation, enabling the model to capture global contextual information. Upon training completion, we combine the one-hop and multi-hop node representations to enhance feature expressiveness and improve spatial domain identification accuracy.

### Data preprocessing and spatial graph construction

In accordance with STAGATE [19], all outlier spots in the ST datasets utilized in this study were removed. Subsequently, the original gene expression data underwent logarithmic transformation and normalization using the SCANPY package [23]. Finally, the top 3000 most highly variable genes were selected as input features for the gene expression profiles in the MAEST framework.

Next, we constructed a spatial adjacency graph utilizing the spatial location information of each spot. We define a graph $ G = (V, A, X) $, where $ V $ represents the set of nodes (i.e. spots) and $ N = |V| $ denotes the number of spots. $ \mathbf{A} \in \{0, 1\}^{N \times N} $ represents the adjacency matrix. $ X \in \mathbb{R}^{N \times d_{in}} $ denotes the input node feature matrix, that is, the preprocessed gene expression profiles. For each spot, its neighbors are defined as the $ k $ nearest points based on spatial location information determined by Euclidean distance. A bidirectional edge exists between each node and its $ k $ neighbors. Based on our evaluations and insights from GraphST [22], we determined that selecting $ k = 3 $ produces optimal results. In summary, we constructed an undirected connected graph using positional information from ST, with the preprocessed gene expression data incorporated as node features.

### Masked feature reconstruction

ST sequencing technology currently faces challenges such as substantial missing values and noise, which can significantly compromise the quality of downstream analyses [24]. Enhancing spatial domain recognition by denoising ST data thus becomes a critical consideration. Denoising autoencoder [25] offers an effective strategy for addressing this issue by intentionally corrupting input data, thereby encouraging the model to avoid learning trivial solutions. Masking, as a corruption technique in autoencoders, has seen widespread success in computer vision (CV) [26, 27] and natural language processing [28]. Inspired by these advancements, graph masked autoencoders have emerged as a promising approach in graph self-supervised learning [29, 30].

Beyond issues of missing values and noise, ST data often contain significant redundancy due to the similar gene expression profiles shared within the same spatial domain. This redundancy reduces the challenge for the encoder, limiting its ability to learn rich and expressive representations. By incorporating a masking strategy, graph masked autoencoders effectively mitigate this redundancy, placing greater emphasis on the decoder to reconstruct the data. This approach helps avoid feature collapse and promotes the learning of more informative representations [29]. In the following, we implement masked feature reconstruction using a graph mask autoencoder. We enhance the encoding and decoding capabilities, as well as the stability of both the encoder and decoder, through multiple random re-masking and model regularization.

#### Masked graph autoencoders

In the graph autoencoder framework, we denote $ f_{E} $ as the encoder and $ f_{D} $ as the decoder. The hidden layer is represented as $ H \in \mathbb{R}^{N \times d} $. The primary objective of the autoencoder is to learn the representation of $ H $ or a well-initialized $ f_{E} $ by reconstructing the features or structure of the input nodes:


(1) \begin{align*}& H = f_{E}(A, X), \quad \widetilde{G} = f_{D}(A, H)\end{align*}

where $ \widetilde{G} $ represents the reconstructed graph characteristics.

In the feature masking process, we sample a subset of nodes $ \tilde{\mathcal{V}} \subset \mathcal{V} $ and mask each of their features with an all-zero $ N $-dimensional vector. Consequently, the node features in the masked feature matrix are defined as


(2) \begin{align*}& \widetilde{x}_{i} = \begin{cases} 0 & \textrm{if } v_{i} \in \tilde{\mathcal{V}} \\ x_{i} & \textrm{if } v_{i} \notin \tilde{\mathcal{V}} \end{cases}\end{align*}

The objective is to reconstruct the masked features of nodes in $ \tilde{\mathcal{V}} $ based on the partially observed node signals $ \widetilde{X} $ and the input adjacency matrix $ A $. Subsequently, the corrupted graph $ (A, \widetilde{X}) $ is input into the encoder to generate representations $ H $. The decoder $ f_{D} $ then decodes from $ H $ to obtain the masked features $ Z $. The training objective is to match the predicted $ Z $ with the original features $ X $ based on a specified criterion.

#### Multiple random re-mask

To enhance the reconstruction of input features, we aim to introduce randomness into the decoding process, necessitating the decoder to recover the input $ X $ from diverse and partial observations of the embeddings [31].

Specifically, before inputting the hidden layer $ H $ into the decoder, we apply multiple random masks to $ H $, resembling the random propagation observed in semi-supervised learning [32]. Analogous to the feature masking of nodes discussed above, we randomly select a subset of nodes $ \tilde{\mathcal{V}} \subset \mathcal{V} $, ensuring that all nodes have an equal probability of being chosen, regardless of prior masking. Subsequently, we set the hidden layer $ H $ of the selected nodes to zero:


(3) \begin{align*}& \widetilde{\boldsymbol{h}}_{i} = \begin{cases} \boldsymbol{0} & \textrm{if } v_{i} \in \overline{\mathcal{V}} \\ \boldsymbol{h}_{i} & \textrm{if } v_{i} \notin \overline{\mathcal{V}} \end{cases}\end{align*}

Subsequently, the decoder reconstructs the input $ X $ from the corrupted $ \widetilde{H} $. This process is repeated $ K $ times. Each view contains distinct information following re-masking, and all views are required to reconstruct the input node features. Finally, we employ the scaled cosine error [29] to quantify the reconstruction error and aggregate the errors from $ K $ views for training:


(4) \begin{align*}& \mathcal{L}_{recon.} = \frac{1}{|\tilde{\mathcal{V}}|} \sum_{j=1}^{K} \sum_{v_{i} \in \widetilde{\mathcal{V}}} \left(1 - \frac{x_{i}^\top z_{i}^{(j)}}{\|x_{i}\| \cdot \|z_{i}^{(j)}\|}\right)^\gamma\end{align*}

where $ x_{i} $ is the $ i $th row of $ X $, $ z_{i}^{(j)} $ is the $ i $th row of the predicted feature $ Z^{(j)} = f_{D}(A, \widetilde{H}^{(j)}) $, and $ \gamma \geq 1 $ is the scaling coefficient. In this study, the decoder, similar to the encoder, is structured as a single-layer GAT [33]. We opt for a single-layer GNN to ensure that nodes receive information exclusively from one-hop neighbors, thereby learning more specific representations and mitigating over-smoothing, which can result in blurred spatial domain boundaries.

#### Model regularization

Throughout the experimental process, we observed that the initialization of model parameters significantly influences subsequent training results. To mitigate the effects of random initialization on training outcomes and to prevent corrupted inputs from disrupting the model, we strive to adjust the model during the early stages of training.

Formally, we use the hidden layer $ H $, derived from inputting the complete unmasked graph into the encoder, as a standard, aiming to ensure that the $ H^{\prime} $ obtained from the corrupted graph predicts it as closely as possible. Specifically, we define a projector $ g $, analogous to the decoder in input feature reconstruction, to map the code $ H $ into the representation space for prediction. During training, the target generator employs the target representation derived from the unmasked graph as $ \bar{X} $. Subsequently, the results from the encoder utilizing the masked graph $ H $ are projected into the representation space, resulting in $ \bar{Z} $:


(5) \begin{align*}& \bar{Z} = g(H), \quad \bar{X} = g(f_{E}(A, X))\end{align*}

We then train the encoder and projector network to minimize the distance between $ \bar{Z} $ and $ \bar{X} $, assessing this using the scaled cosine error:


(6) \begin{align*}& \mathcal{L}_{reg.} = \frac{1}{N} \sum_{i=1}^{N} \left(1 - \frac{\bar{z}_{i}^\top \bar{x}_{i}}{\|\bar{z}_{i}\| \cdot \|\bar{x}_{i}\|}\right)^\gamma\end{align*}

The projector is implemented as a multi-layer perceptron (MLP). During the initial epochs, the $ \mathcal{L}_{reg.} $ significantly impacts the model, exerting a corrective effect. However, due to the rapid fitting capabilities of the MLP, $ \mathcal{L}_{reg.} $ quickly approaches zero, preventing it from influencing the model’s stability in later training stages.

### Node discrimination

In graph self-supervised learning, both graph masking models and graph contrastive learning models are leading approaches for achieving optimal performance. Unlike graph masking autoencoders, graph contrastive learning generates positive and negative sample pairs from the same graph through data augmentation techniques. The objective is to minimize the distance between positive pairs while maximizing the distance between negative pairs, which helps prevent model collapse and ensures that the learned low-dimensional representations are well-distributed in the feature space [34]. Recent studies suggest that contrastive learning tends to focus on global information, whereas masking models excel at capturing local patterns [35]. Therefore, combining contrastive learning with masking models has the potential to mitigate the weaknesses of each approach, as shown in CV applications [36]. Building on this insight and informed by established graph contrastive learning models [37, 38], we have incorporated a novel node discrimination module into the original MAEST framework. This module enhances the model’s ability to capture both global and local information, leading to improved performance in spatial domain detection.

Starting with the original attribute graph $ G $, we first generate an augmented view graph $ G^{\prime} $ using graph data augmentation $ \tau $, constructed from $ X^{\prime} $ and $ A^{\prime} $. Specifically, without modifying the original graph’s topology, we construct the augmented graph by randomly permuting the gene expression vectors among the nodes. Next, we employ an encoder $ f_{E} $ with parameter sharing from the Masked graph autoencoders to derive the latent embeddings $ H, H^{\prime} \in \mathbb{R}^{N \times d} $ for both the original and augmented graphs. Then, a neural network projection head $ p $ with shared parameters maps the node embeddings to a new latent space, where the self-supervised learning loss will be applied. This outputs $ Z $ and $ Z^{\prime} \in \mathbb{R}^{N \times d^{\prime}} $, representing the projection results for the original and augmented data, respectively.


(7) \begin{align*}& H^{\prime} = f_{E}(A^{\prime}, X^{\prime}), \quad Z^{\prime} = p(H^{\prime})\end{align*}

Finally, we aggregate the new node embeddings into node summaries $\mathbf{g}, \mathbf{g}^{\prime} \in \mathbb{R}^{N \times 1}$ using a feature aggregation method. We train the encoder $ f_{E} $ and the projection head $ p $ by minimizing the binary cross-entropy loss function, which distinguishes between the original and augmented node summaries [38]. The node discrimination loss is shown in Equation 8.


(8) \begin{align*}& \begin{aligned} \mathcal{L}_{\mathrm{discri.}} &= \frac{1}{N} \sum_{i=1}^{N} \left(1 \cdot \log \frac{1}{\mathbf{g}_{i}} + 0 \cdot \log \frac{1}{1 - \mathbf{g}_{i}}\right) \\ & \quad + \frac{1}{N} \sum_{i=1}^{N} \left(0 \cdot \log \frac{1}{\mathbf{g}_{i}^{\prime}} + 1 \cdot \log \frac{1}{1 - \mathbf{g}_{i}^{\prime}}\right) \\ &= \frac{1}{N} \sum_{i=1}^{N} \left(\log \frac{1}{\mathbf{g}_{i}} + \log \frac{1}{1 - \mathbf{g}_{i}^{\prime}}\right) \end{aligned}\end{align*}

where the first component reduces the loss associated with classifying original node summary embeddings as category 1, while the second component reduces the loss for augmented node summary embeddings classified as category 0.

In summary, the encoder is trained by maximizing the mutual information between the graph’s high-level “global” representation and the “local” representation of the input nodes, while simultaneously minimizing the mutual information between the representations of negative node-graph pairs [21]. This training strategy encourages the encoder to capture globally relevant information, such as spatial domain category labels. The goal of the node discrimination module is not to make all nodes exhibit similar features, but rather to ensure that nodes within the same spatial domain or with similar expressions are more similar to each other.

### Overall loss function

The representation learning component of MAEST is trained by minimizing the reconstruction loss, regularization loss, and node discrimination loss. The overall loss can be briefly defined as


(9) \begin{align*}& \mathcal{L} = \mathcal{L}_{recon.} + \lambda_{1} \mathcal{L}_{reg.} + \lambda_{2} \mathcal{L}_{\mathrm{discri.}}\end{align*}

Here, $\lambda _{1}$ and $\lambda _{2}$ are coefficients for the model regularization and node discrimination losses, respectively, used to balance their effects. By default, $\lambda _{1}$ and $\lambda _{2}$ are set to 0.2 and 0.02, respectively, with a learning rate of 0.001 and 900 epochs.

### Integrating multi-hop information

To enhance clustering results, it is essential to ensure that the processed data exhibit expression features that are as similar as possible within the same spatial domain while remaining as dissimilar as possible across different spatial domains. This objective aligns with a fundamental clustering principle: maximizing intra-class similarity while minimizing inter-class similarity. This necessitates that nodes possess features unique to their respective classes. Research indicates that deeper GNNs can capture more complex higher-order graph structures, thereby enhancing the discriminative ability for minority nodes [39]. However, increasing the depth of GNNs may also lead to feature over-smoothing among nodes.

To preserve the integrity of learned node features while incorporating higher-order graph structures, we have designed an integrated multi-hop information module. We utilize the hidden layer $ H $ from the previous context as input for this module. This layer is then processed through an $ n $-layer GNN network $ f_{n} $, which contains only an aggregation module without learnable parameters, to obtain embeddings $ H_{D} $ that capture deep graph structures. The final output, $ H_{out} $, defined as the sum of $ H $ and $ H_{D} $, is subsequently used as input for downstream clustering tasks:


(10) \begin{align*}& H_{D} = f_{n}(H), \quad H_{out} = H + H_{D}\end{align*}

Here, $ n $ is set to 3 by default.

### Clustering

We utilized the mclust [40] algorithm to cluster the processed embeddings (as shown in Fig. 1B), assigning each point to distinct spatial domains [41, 42]. Each cluster consists of spots exhibiting similar gene expression and proximity in spatial location, thus considered part of the same spatial domain. For tissue slices with manual annotations, we set the number of clusters to correspond with the actual values. For tissue slices without manual annotations, we utilized annotations from comparative methods and the histological characteristics of the slices to guide our selection of the cluster count, ensuring alignment with the cluster numbers used by comparable approaches.

### Data description

We utilized five spatial transcriptomic gene expression datasets in our study (see Supplementary Table S2 for details). Below, we describe each dataset:

1. **LIBD human dorsolateral prefrontal cortex (DLPFC)**:
  This dataset includes 12 tissue slices obtained using the 10x Visium platform [43] (http://research.libd.org/spatialLIBD/). Each slice comprises between 3460 and 4789 spots, collectively capturing 33 538 genes. Manual annotations divide each slice into five to seven layers, corresponding to the DLPFC layers and white matter (WM).
2. **Mouse olfactory bulb**:
  Sequenced using Stereo-seq technology, this dataset was processed and annotated by Dong et al. [19]. It contains 19 109 spots, each profiling 14 376 genes.
3. **Mouse hippocampus**:
  Generated using Slide-seqV2 technology, this dataset originates from section *Puck_200115_08* (https://portals.broadinstitute.org/single_cell/study/slide-seq-study). It consists of 52 869 spots.
4. **Mouse organogenesis spatiotemporal transcriptomic atlas (MOSTA)**:
  This dataset, sequenced with Stereo-seq technology, is accessible at https://db.cngb.org/stomics/mosta/. We used four sections representing different developmental stages: E11.5, E12.5, E13.5, and E14.5. Spot counts range from 30 124 to 92 928 across these sections, with gene counts varying from 18 566 to 27 810.
5. **Mouse brain tissue**:
  This dataset, sourced from the 10x Genomics Data Repository (https://www.10xgenomics.com/datasets), comprises anterior and posterior sections. Each section contains between 1868 and 3289 spots, capturing 32 285 genes. Horizontal integration data for the anterior and posterior sections were derived from Long et al. [22].

These diverse datasets, covering a wide range of spatial transcriptomic technologies, sample types, and biological contexts, provide a comprehensive basis for evaluating the performance of our MAEST framework.

## Results and discussion

### Overview of MAEST

MAEST is a deep GNN framework (Fig. 1) that embeds both spatial location and gene expression profiles into a latent feature space. By integrating graph-masked autoencoders with graph contrastive learning, MAEST improves both model robustness and representational capacity. Additionally, it incorporates one-hop and multi-hop node information to enhance feature expressiveness. Experimental results demonstrate that the integrated features generated by MAEST outperform those of state-of-the-art methods in downstream spatial domain clustering tasks.

MAEST constructs a spatial adjacency graph based on the relative positions of spatial spots, using gene expression profiles as node features. During the training phase (Fig. 1A), MAEST incorporates two core tasks: masked feature reconstruction and node discrimination, leveraging the latent representations of a masked graph and a shuffled graph generated by a shared encoder, respectively.

For the *masked feature reconstruction* task, the masked graph is created by retaining the original graph’s adjacency matrix while randomly masking a portion of the node features. This masked graph is then encoded and decoded, with the objective of reconstructing the original features of the masked nodes. To enhance the encoder’s representational capacity, multiple re-masking processes are applied to the latent representations, allowing the model to reconstruct the graph from diverse perspectives. To ensure stability, a regularization component is introduced: an MLP maps the latent representation of the masked graph back to the original graph’s latent representation, enforcing proximity between masked and original features.

For the *node discrimination* task, the shuffled graph is generated by retaining the original adjacency matrix while randomly shuffling the node features. Both the original and shuffled graphs are encoded by the shared GNN encoder, and their representations are further compressed using a shared MLP to obtain low-dimensional embeddings. Positive and negative pairs are then generated for contrastive learning. Positive pairs align node features from the original graph with its global representation, while negative pairs align node features from the original graph with the global representation of the shuffled graph. This contrastive learning framework ensures that node embeddings are aligned with the context of the original graph and distinct from the corrupted graph, promoting a uniform distribution of features in the latent space.

By combining these tasks, MAEST enhances the robustness and expressiveness of its learned representations. The final embedding is generated by merging the one-hop representation with multi-hop representations, effectively capturing both local and long-range spatial dependencies (Fig. 1B). This approach enables the model to differentiate distant spots with similar expression patterns. Finally, the Mclust algorithm is employed to identify spatial domains.

### MAEST improves accuracy of spatial domain recognition in human dorsolateral prefrontal cortex dataset

To quantitatively assess MAEST’s performance in spatial domain identification, we applied it to the LIBD human DLPFC dataset. This dataset contains spatially resolved transcriptomic profiles from 12 DLPFC slices, each representing four to six layers of the cortex and the WM region. We compared MAEST against seven recently proposed spatial clustering methods: GraphST [22], STAGATE [19], DeepST [18], CCST [20], BayesSpace [14], SpaGCN [17], and Giotto [44]. Clustering performance was evaluated using three metrics: accuracy (ACC), adjusted rand index (ARI) [45], and normalized mutual information (NMI) [46].

**Figure 2:**
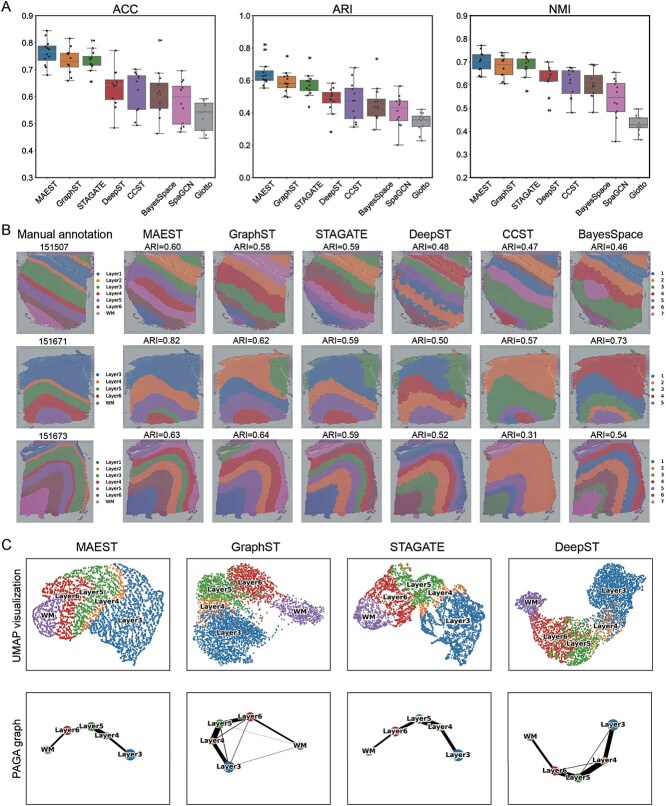
MAEST enhances the spatial domain recognition in human DLPFC dataset. (A) Boxplots showing the accuracy (ACC), ARI, and NMI scores for the eight methods applied to the 12 DLPFC slices. The center line in each boxplot represents the median, the box limits indicate the upper and lower quartiles. (B) Clustering result visualizations for slices 151507, 151671, and 151673 of the DLPFC dataset, comparing MAEST with GraphST, STAGATE, DeepST, CCST, and BayesSpace. (C) UMAP visualizations and PAGA trajectory inference results generated from the embeddings of MAEST, GraphST, STAGATE, and DeepST on slice 151671 of the DLPFC dataset.

Across all 12 slices, MAEST achieved the highest median scores on all three evaluation metrics (ACC = 0.77, ARI = 0.62, and NMI = 0.71), outperforming the other methods. Notably, MAEST also exhibited smaller variations in clustering results across different slices compared to most other approaches (Fig. 2A). Among the comparison methods, GraphST and STAGATE performed similarly, with GraphST achieving median scores (ACC = 0.73, ARI = 0.59, and NMI = 0.69), and STAGATE scoring (ACC = 0.73, ARI = 0.58, and NMI = 0.69), both slightly trailing MAEST. The remaining five methods demonstrated a more pronounced performance gap compared to the top three.The clustering evaluation results of each slice refer to Supplementary Table S3.

Next, we visualized the clustering results of the top six methods on three slices: 151507, 151671, and 151673 (Fig. 2B). In slice 151507, all six methods generally captured the relative layer structure. MAEST and STAGATE were closest to the manual annotations, though layer 2 was not finely delineated. GraphST struggled to accurately depict the thickness of the layers, completely missing layer 4. While DeepST and CCST identified layer 2 with greater precision, their overall performance was average, as they failed to clearly distinguish layers 3 through 6. BayesSpace performed poorly, with only layer 1 being relatively accurately positioned, while the other layers were inadequately separated. In slice 151671, all methods had difficulty identifying the narrow and indistinct layer 4. Among them, MAEST, CCST, and BayesSpace successfully identified the large spatial domain of layer 3, while the remaining methods incorrectly divided it into two separate domains. In slice 151673, five of the six methods, except for CCST, accurately identified the stratification of the cortex. MAEST, GraphST, and STAGATE produced similar results, effectively capturing the overall layer hierarchy, though layers 2 and 4 were depicted as thicker than indicated in the annotations. DeepST showed a significant discrepancy with the annotations in layers 3 and 6. BayesSpace incorrectly divided the WM region into two distinct spatial domains. In conclusion, the results demonstrate that MAEST produces more accurate domain identification results and performs particularly well in maintaining consistency within large spatial domains. Results for the remaining slices can be found in Supplementary Fig. S1.

MAEST integrates spatial specificity with both local and global information of spots, generating a more expressive representation. This enhanced representation is visualized using UMAP [47], which effectively captures the relative locations of different spatial domains, providing a clear depiction of spatial trajectories. For instance, in slice 151671 (Fig. 2C, upper), the UMAP plot generated through MAEST embedding accurately restores the relative positions of spots and spatial domains, with clearly defined boundaries among different spatial domains (refer to the slice annotations in Fig. 2B). We compared the UMAP results of MAEST with those from the top three comparison methods. The UMAP plots produced by GraphST and STAGATE can roughly illustrate the relative positions of spatial domains; however, neither method effectively shows the relative positions of individual spots. Specifically, GraphST’s UMAP exhibits slight mixing in layers 4 to 6, while DeepST’s UMAP does not distinctly separate the spots in these layers. Subsequently, we employed the trajectory inference algorithm PAGA [48] to validate the inferred trajectories (Fig. 2C, lower). The PAGA plots generated by MAEST and STAGATE demonstrate a nearly linear developmental trajectory from layer 3 to layer 6 and the WM, as well as the similarities between adjacent layers. In contrast, the PAGA results produced by GraphST and DeepST indicate a certain degree of mixing between different layers.

### MAEST enables finer-grained spatial domain identification in high-resolution Slide-seqV2 and Stereo-seq spatial transcriptomics

**Figure 3:**
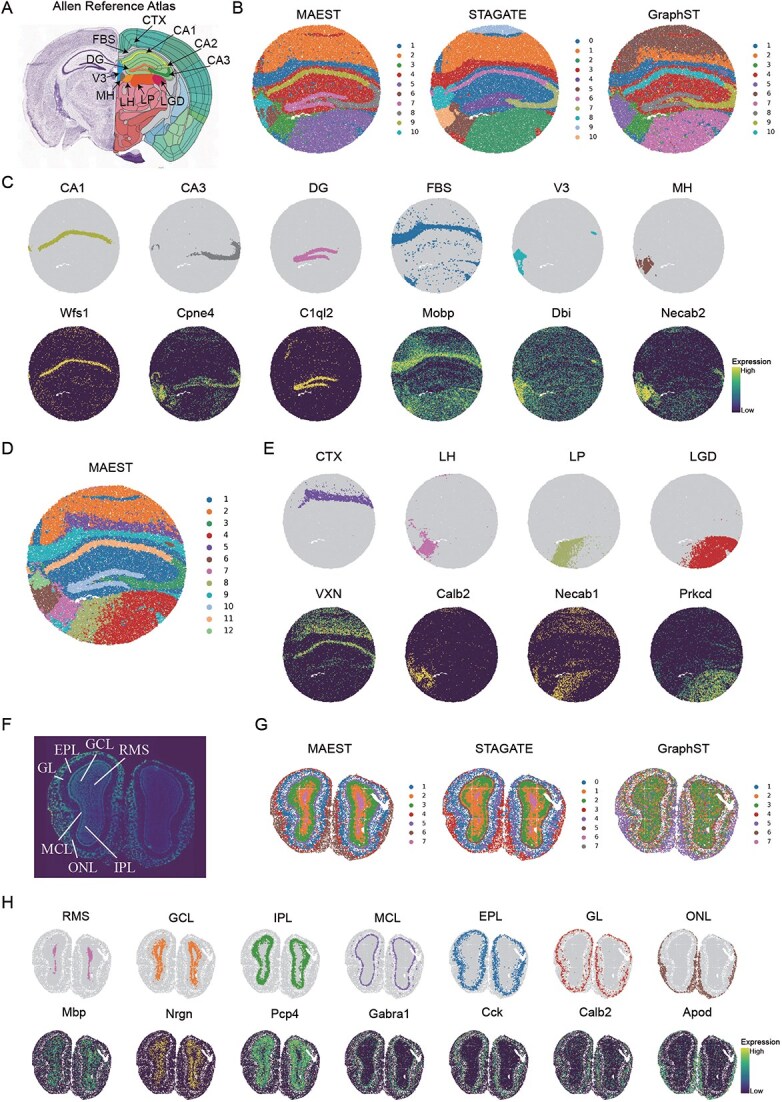
MAEST enhances the recognition of known structures in mouse hippocampus and olfactory bulb tissues. (A) Annotations of hippocampal regions from the Allen Mouse Brain Atlas. (B) Spatial domains identified by MAEST, STAGATE, and GraphST in mouse hippocampus tissue acquired by Slide-seq V2. (C) Visualization of spatial domains identified by MAEST in mouse hippocampus tissue and the corresponding expression patterns of marker genes. (D) Clustering results of MAEST on mouse hippocampus data with a finer-grained. (E) Visualization of the finer-grained spatial domains (CTX, LH, LP, and LGD) and their corresponding marker genes identified. (F) Laminar organization of the mouse olfactory bulb annotated in the DAPI-stained image generated by Stereo-seq. (G) Spatial domains identified by MAEST, STAGATE, and GraphST in the mouse olfactory bulb Stereo-seq dataset. (H) Visualization of spatial domains identified by MAEST in mouse olfactory bulb tissue and the corresponding expression patterns of marker genes.

We subsequently compared MAEST, GraphST, and STAGATE using the mouse hippocampus dataset obtained via high-resolution Slide-seqV2 technology [8]. For this analysis, we employed the annotated Allen Brain Atlas as the ground truth reference (Fig. 3A). All three methods successfully identified the main anatomical regions, including the forebrain bundle system (FBS), the dentate gyrus, and the pyramidal layers within Ammon’s horn, which can be further divided into fields CA1, CA2, and CA3. Moreover, MAEST accurately captures both the location and shape of the third ventricle (V3) and its adjacent medial habenula (MH). Although STAGATE is capable of identifying the location of V3 and MH, it exhibits a significant discrepancy in shape compared to the anatomical annotations. In contrast, GraphST merges V3 and MH into a single spatial domain.

We compared the distribution of marker genes with the corresponding domains identified by MAEST to further validate its clustering results (Fig. 3C). Most clustering regions aligned well with the corresponding marker genes. Specifically, MAEST accurately delineated the shapes of V3 and MH regions based on the expression of *Dbi* and *Necab2* genes, which closely align with their anatomical structures. The *Mobp* and *Dbi* genes, while predominantly expressed in the FBS and V3 regions respectively, also show trace expression in other areas, contributing to the stability of myelin sheaths and the development of the nervous system [49, 50].

We observed that all methods struggled to distinguish the lateral posterior nucleus of the thalamus (LP) from the dorsal part of the lateral geniculate complex (LGD) at the original resolution. To achieve finer-grained spatial domain identification, we increased the detection resolution by raising the number of clusters from 10 to 12 (Fig. 3D). As a result, MAEST successfully separated the LP and LGD regions and accurately identified the laminar structure of the cerebral cortex (CTX). In contrast, increasing the number of clusters for STAGATE and GraphST resulted in only minimal improvements in clustering performance (Supplementary Fig. S2). We further visualized MAEST’s clustering results in the CTX, lateral habenula (LH), LP, and LGD regions, which closely aligned with the spatial distribution of their corresponding marker genes (Fig. 3E). LP functions as a relay station for sensory information, facilitating both its transmission and processing [51]. LGD serves as a relay nucleus in the visual system, primarily responsible for processing and transmitting visual signals [52]. Although these regions share functional and locational similarities, they also exhibit distinct differences. These findings demonstrate the expressiveness of the latent representations learned by MAEST, underscoring its ability to capture sub-domains with fine granularity.

We next applied MAEST to a coronal mouse olfactory bulb dataset obtained via Stereo-seq [4] to evaluate its ability to resolve fine-grained spatial domains in the mouse brain. Manual annotations of laminar organization based on DAPI-stained images [53] served as a reference. These annotations include the rostral migratory stream (RMS), granule cell layer (GCL), internal plexiform layer (IPL), mitral cell layer (MCL), external plexiform layer (EPL), and olfactory nerve layer (ONL) (Fig. 3F).

The clustering results from MAEST, STAGATE, and GraphST revealed the hierarchical structures of the mouse olfactory bulb but with varying levels of granularity (Fig. 3G). GraphST was the least effective, identifying only broad inner and outer layers without clearly distinguishing specific sub-layers. In contrast, both MAEST and STAGATE captured distinct hierarchical structures. MAEST accurately separated the ONL, GCL, and EPL layers in the outer regions, whereas STAGATE identified only two layers, failing to differentiate the GCL and EPL regions. In the inner layers, both methods successfully distinguished the RMS, GCL, and IPL. However, MAEST provided a much clearer separation of the GCL and IPL domains, while STAGATE exhibited a noticeable overlap between these layers.

To assess the biological relevance of MAEST’s clustering results, we compared them to the spatial expression patterns of region-specific marker genes (Fig. 3H). MAEST’s clusters closely matched the anatomical regions defined by marker gene expression. While certain marker genes, such as *Mbp* and *Gabral*, showed some overlap with neighboring regions due to boundary effects, MAEST effectively delineated the major anatomical regions of the mouse olfactory bulb. This demonstrates its ability to resolve sub-domains with fine granularity, even in complex and high-resolution datasets.

### MAEST excels in deciphering spatial domains in mouse embryo development

**Figure 4:**
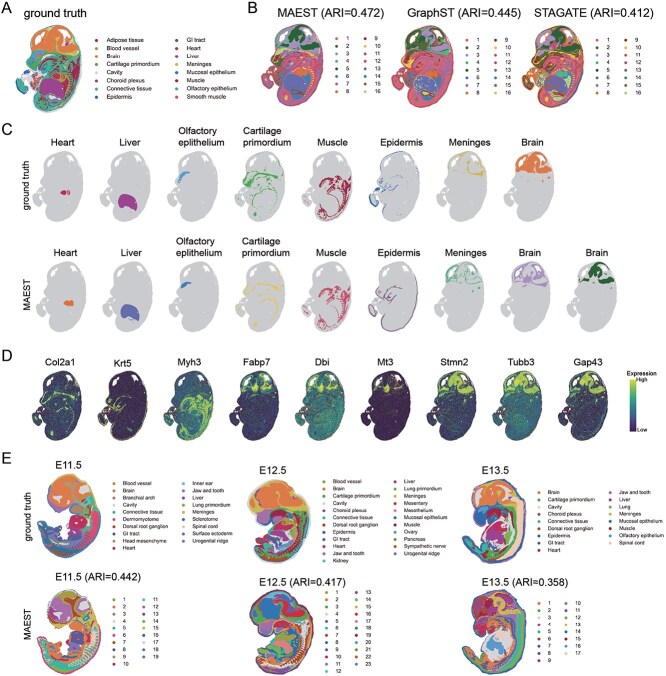
MAEST accurately identifies spatial domains in mouse embryo tissues. (A) Ground truth annotations of the E14.5 stage mouse embryo dataset. (B) Spatial clustering results of the E14.5 stage mouse embryo generated by MAEST, GraphST, and STAGATE. (C) Key domains identified by MAEST compared with the corresponding ground truth annotations. (D) Visualization of marker genes on different tissues of the E14.5 mouse embryo. (E) Spatial domain identification results from MAEST for E11.5, E12.5, and E13.5 stages of mouse embryos.

To further evaluate MAEST’s performance in complex biological scenarios, we applied it to the mouse embryo Stereo-seq dataset, focusing on its ability to resolve spatial domains within embryonic tissues. Our analysis began with the E14.5 stage mouse embryo dataset, encompassing 92 928 cells and 18 566 genes [4] (Fig. 4A).

We compared the clustering results of MAEST with those of GraphST and STAGATE at the E14.5 developmental stage (Fig. 4B), and the ARI values of their clustering results were 0.472, 0.445, and 0.412, respectively. All three methods identified the structure and spatial distribution of major tissues and organs, including the heart, liver, cartilage primordium, muscle, and brain. However, MAEST stood out by providing more precise boundaries and consistent clustering of these regions, outperforming both alternatives.

To validate MAEST’s performance, we aligned its results with annotated tissue and organ references (Fig. 4C, Supplementary Fig. S3). The domains identified by MAEST closely matched the ground truth annotations, as supported by the expression patterns of key marker genes such as *Col2a1*, *Krt5*, and *Myh3* (Fig. 4D), which correspond to the cartilage primordium, epidermis, and muscle, respectively. Notably, MAEST’s clustering of the liver region produced a cohesive domain consistent with annotations, while GraphST and STAGATE exhibited significant spot mixing. In the brain, although both MAEST and GraphST provided relatively complete clustering, only MAEST successfully identified the olfactory epithelium as a distinct region. Moreover, MAEST delineated the epidermis with no intermixing of other domains, unlike its counterparts.

Interestingly, MAEST divided the brain region of the E14.5 mouse embryo into two distinct subdomains (Fig. 4C). Marker gene analysis revealed distinct gene expression profiles between these regions (Fig. 4D). The first region, characterized by the expression of genes like *Fabp7*, *Dbi*, and *Mt3*, predominantly included glial cells, particularly astrocytes, and was associated with neuron support, lipid metabolism, and glial regulation [50, 54]. The second region, enriched with *Stmn2*, *Tubb3*, and *Gap43*, was linked to neuronal cell functions, including growth, repair, and plasticity [55, 56]. By distinguishing these regions, MAEST revealed functional specialization within the brain, showcasing its ability to uncover intricate cellular architectures and biological insights.

Finally, we extended our analysis to mouse embryo datasets from developmental stages E11.5, E12.5, and E13.5 (Fig. 4E). Across all stages, MAEST effectively reconstructed the spatial domain structures, accurately identifying major organs such as the brain, heart, and lungs, as well as tissues like the sclerotome and muscle. This robust performance across multiple developmental stages highlights MAEST’s reliability in processing large-scale spatial transcriptomic data. The specific locations of each spatial domain identified by MAEST and the reference comparison with the ground truth are shown in Supplementary Figs S4–S6.

In summary, MAEST not only excels at identifying spatial domains in embryonic tissue but also provides fine-grained resolution of structural and functional details. Its superior clustering performance underscores its utility in studying complex tissue development and cellular specialization.

### MAEST facilitates consistent horizontal integration across adjacent tissue slices

**Figure 5:**
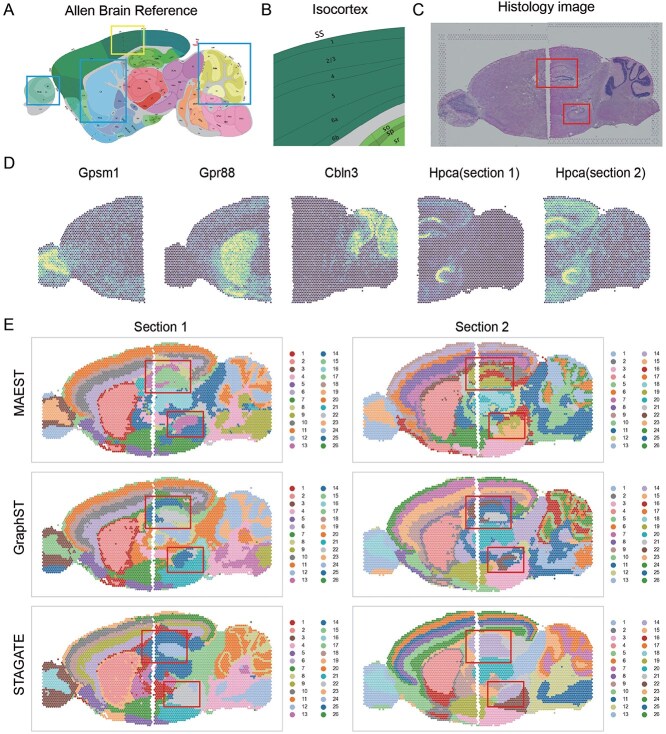
MAEST facilitates horizontal integration of ST data across anterior and posterior slices of the mouse brain. (A) Annotations from the Allen Brain Atlas for a sagittal section of the mouse brain, used as a reference for spatial domain identification. (B) Annotation of the Isocotex area in Allen Brain Atlas. (C) Histological images of the anterior and posterior slices of the mouse brain, highlighting key anatomical structures. (D) Spatial expression patterns of marker genes (*Gpsm1*, *Graph88*, *Cbln3*, *Hpca*) in the anterior and posterior slices. (E) Horizontal integration results for two mouse brain samples, each comprising anterior and posterior slices. Spatial domain identifications are presented from top to bottom for MAEST, GraphST, and STAGATE, illustrating their respective clustering performances.

ST technologies often capture only a fraction of a tissue sample, necessitating the division of larger tissues into multiple horizontal slices, each sequenced independently. Horizontal integration allows researchers to merge data from these sections to achieve a cohesive view of the entire tissue. To evaluate the horizontal integration capabilities of MAEST, GraphST, and STAGATE, we utilized two groups of 10X Visium mouse brain datasets, each divided into anterior and posterior slices. We aligned the anterior and posterior slices using SpaGCN. Using annotations from the Allen Brain Atlas as the reference (Fig. 5A), we compared the integration results across the datasets.

We assessed clustering performance for each section independently (Fig. 5E). All three methods successfully reconstructed the main spatial domains of the mouse brain, including the olfactory bulb, caudoputamen, and cerebellar cortex (teal boxes in Fig. 5A). The clustering results were validated using the expression patterns of marker genes such as *Gpsm1*, *Graph88*, and *Cbln3* (Fig. 5D), confirming the accuracy of domain identification. The relatively poor results of SpaGCN can be found in Supplementary Fig. S7.

Horizontal integration was further evaluated based on the isocortex, a region that comprises five sequential layers: layer 1, layer 2/3, layer 4, layer 5, and layers 6a and 6b (yellow boxes in Fig. 5A and B). Both MAEST and GraphST consistently identified the five-layer structure and maintained coherence across slice junctions (Fig. 5E). However, STAGATE exhibited inconsistencies, splitting layer 2/3 vertically at the slice boundary in Section 1. In Section 2, MAEST again successfully identified all five layers, while GraphST merged layers 4 and 5 into a single domain. STAGATE showed an improved performance by distinguishing layers 6a and 6b but continued to exhibit artifacts in other regions (Fig. 5E).

In the middle regions of the slices, significant differences emerged. STAGATE’s results suffered from over-smoothing, failing to capture detailed structures such as the dorsal (top) and ventral (bottom) horns of the hippocampus (highlighted by red boxes in Fig. 5C and E). In contrast, both MAEST and GraphST accurately identified these regions, particularly the dorsal region, which spans two slices. Notably, MAEST outperformed GraphST in aligning its clustering results with the spatial expression distribution of the *Hpca* gene (Fig. 5D), a key marker for pyramidal cells in the hippocampal CA1 region [57].

MAEST demonstrated superior horizontal integration capabilities, effectively merging adjacent tissue slices while preserving structural continuity and accurately representing internal details. By providing consistent spatial domain identification and capturing fine structural nuances, MAEST outperforms GraphST and STAGATE in reconstructing a cohesive view of ST data.

### Ablation study

To evaluate the contributions of individual components and hyperparameters in MAEST, we conducted ablation studies using four sections (151669–151672) from the DLPFC dataset, which include manual annotations. The ARI was employed as the metric for clustering performance in these experiments.

#### Impact of dropout rates on spatial domain identification

**Figure 6:**
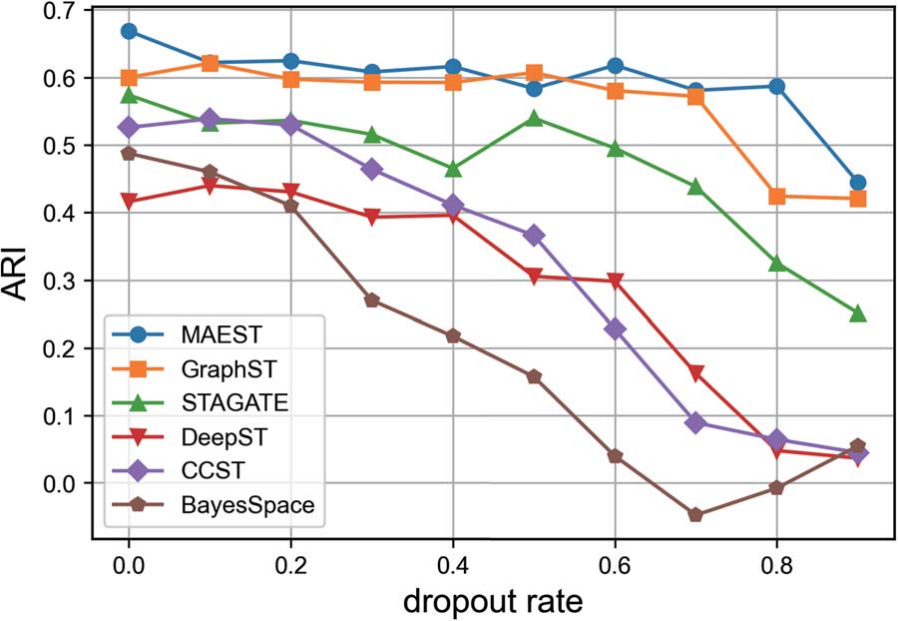
The average clustering performance of MAEST and five comparison methods varies with the dropout rate across four slices (151669–151672) of the DLPFC dataset.

We further evaluated the spatial domain recognition performance of MAEST and five comparison methods using ST data with varying dropout rates (Fig. 6). Here, we randomly zeroed out the data from four sections (151669-151672) of the DLPFC dataset to simulate different proportions of dropout scenarios. The dropout rate was varied from 0 to 0.9, and the average ARI across the four slices was used as the performance metric.

The results indicate that MAEST exhibits high stability across dropout rates ranging from 0 to 0.8, but its performance deteriorates sharply at a dropout rate of 0.9, where it fails to reconstruct the original data. In comparison, GraphST shows slightly lower performance than MAEST, with a marked decline in accuracy starting at a dropout rate of 0.8. STAGATE lags behind the first two methods, with a noticeable decrease in accuracy when the dropout rate reaches 0.6. The remaining three methods demonstrate poor performance, with clustering accuracy declining substantially as the dropout rate increases. The boxplots of the clustering results for each method can be found in Supplementary Fig. S8.

In summary, MAEST not only achieves better clustering results, but also maintains a high degree of stability in the case of high data dropout rates.

#### Impact of model components on clustering accuracy

**Figure 7:**
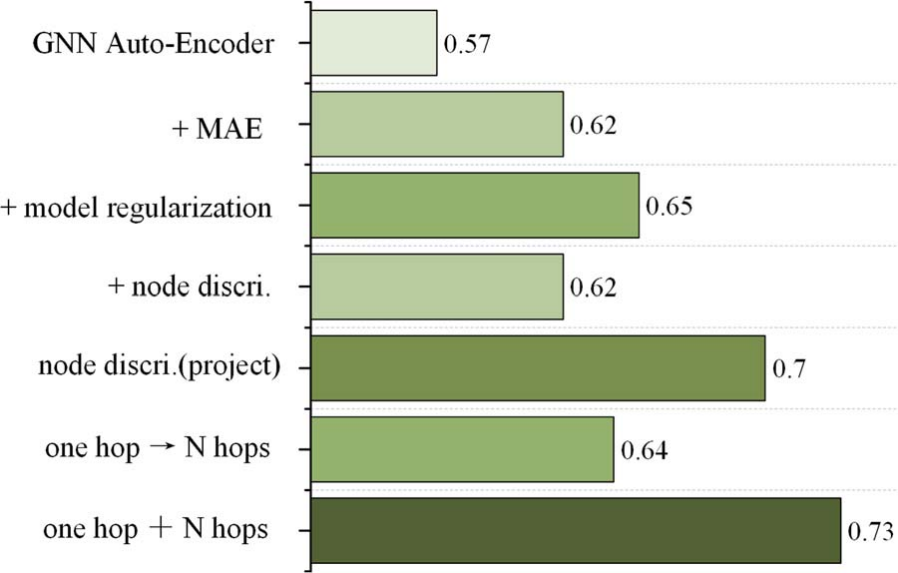
Ablation study of MAEST on slices 151669–151672 of the DLPFC dataset.

We began with a GNN Auto-Encoder as the baseline and incrementally added components to analyze their contributions (Fig. 7). The average ARI across the four slices was used as the performance metric.

- **Baseline performance**: The GNN Auto-Encoder alone yielded the lowest performance, with an average ARI of 0.57.
- **Masked learning**: Adding masked learning improved the ARI to 0.62, as it enhanced node feature representation by leveraging missing data reconstruction.
- **Model regularization**: Incorporating model regularization further boosted the ARI to 0.65 by improving feature stability and convergence.
- **Node discrimination module**: Introducing the node discrimination module initially reduced performance due to high-dimensional features overwhelming the discrimination task. However, applying dimensionality reduction enabled the module to focus on salient features, increasing the ARI to 0.70.
- **Multi-hop integration**: Transitioning from one-hop to multi-hop (N=3) aggregation caused performance degradation due to over-smoothing. By combining one-hop and multi-hop information, the model captured both local and global characteristics, achieving the highest ARI of 0.73.

In summary, each component played a critical role in enhancing clustering accuracy, with their synergistic integration delivering the best results.

#### Effect of mask rate on node representation

We explored the effect of varying mask rates on node representation capability (Supplementary Fig. S9A). Using the one-hop baseline for comparison, we evaluated clustering performance based on final representations combining one-hop and multi-hop features.

- **Optimal mask rate**: Clustering accuracy improved as mask rates increased, reaching a peak when balancing sufficient masking to enhance learning without losing critical features.
- **Excessive masking**: Beyond an optimal threshold, performance declined due to excessive masking, which likely hindered the model’s ability to reconstruct meaningful node representations.

This analysis highlights the importance of tuning mask rates to optimize node feature representation.

#### Impact of multi-hop information on clustering

The role of multi-hop information was further examined (Supplementary Fig. S9B). We compared the clustering effectiveness of representations with varying numbers of hops.

- **Performance trend**: Clustering accuracy improved as the number of hops increased, peaking at four hops. Incorporating long-range node information enriched feature representation.
- **Over-smoothing**: Beyond four hops, the effectiveness diminished as prolonged information aggregation led to over-smoothing, reducing the distinctiveness of node features.

Thus, while multi-hop information is beneficial, careful calibration is necessary to prevent over-smoothing and preserve feature differentiation.

We also examined the impact of varying the weights of $\lambda _{1}$ and $\lambda _{2}$ in the loss function on clustering performance. Overall, both $\lambda _{1}$ and $\lambda _{2}$ exhibit an optimal range of values (Supplementary Fig. S9C,D). The clustering performance is most effective when $\lambda _{1}$ is around 0.1 and $\lambda _{2}$ is approximately 0.01. To further validate the quality of the representations learned by MAEST, we applied clustering algorithms (k-means, Louvain, and mclust) to the representations. While the results from k-means(median ARI = 0.51) and Louvain(median ARI = 0.57) were slightly inferior to those of mclust(median ARI = 0.62), the overall clustering performance surpassed most of the comparison methods (Supplementary Fig. S10). To assess the robustness of the MAEST model, we conducted tests with 40 random seeds (Supplementary Fig. S11). Although some fluctuations were observed in the results for individual slices, the overall performance remained highly stable. For the vast majority of seeds, the median ARI index of the spatial domain identification results of the DLPFC dataset is around 0.62.

The ablation studies demonstrated that each component and hyperparameter in MAEST contributes to its overall clustering accuracy. By combining optimized modules and parameters, MAEST achieves robust performance in spatial domain identification.

## Conclusions

ST has emerged as a transformative sequencing technology that preserves spatial location information while quantifying gene expression, enabling significant advancements in fields such as developmental biology, neuroscience, cancer research, and drug development [1, 3, 6]. A central application of this technology is *spatial domain identification*, which elucidates tissue organization and function by defining distinct biological regions within complex tissues. This task primarily relies on clustering methods that integrate gene expression profiles with spatial data. However, existing approaches often fail to fully harness the unique attributes of ST, resulting in limited representational capacity and suboptimal clustering accuracy.

In this study, we introduced a novel GNN model, MAEST, designed to address these limitations by leveraging the distinctive features of ST data. During training, MAEST employs graph masked autoencoders to denoise and refine spatial data, improving the quality of spot representations. Furthermore, graph contrastive learning is incorporated to mitigate feature collapse and enhance the robustness of embeddings. To stabilize training, regularization techniques are applied during the early stages. Finally, MAEST integrates one-hop and multi-hop representations during clustering, enabling the model to capture both local and global spatial relationships, thereby enhancing clustering precision.

Our experimental results demonstrate that MAEST achieves state-of-the-art performance across diverse ST datasets. Benchmark evaluations on 12 slices from the DLPFC dataset show that MAEST outperforms seven leading methods across multiple metrics. Additionally, MAEST effectively identified spatial domains at various scales in datasets from the mouse hippocampus, olfactory bulb, and embryonic development. In horizontal integration experiments, MAEST demonstrated its ability to seamlessly align and integrate multi-slice data, identifying both slice-specific domains and joint domains across anterior and posterior slices of the mouse brain. Ablation studies confirmed the individual contributions of MAEST’s components, while sensitivity analyses underscored the robustness, effectiveness, and rationality of our approach.

Despite these advances, MAEST faces challenges in identifying smaller spatial domains, particularly at tissue boundaries. The inherent low discriminability of data at these boundaries complicates the precise delineation of spatial domain edges. Future work will focus on developing enhanced techniques to improve the resolution and accuracy of identifying marginal and small-scale spatial domains. In conclusion, MAEST represents a step forward in spatial domain identification, providing a powerful and flexible framework for analyzing ST data across a wide range of biological contexts.

**Key Points**

- This study proposed MAEST, which enhances spatial domain identification by using graph-masked autoencoders for data denoising and refinement, combined with graph contrastive learning to prevent feature collapse and improve model robustness.
- By integrating one-hop and multi-hop representations, MAEST effectively captures both local and global spatial relationships, thereby improving clustering accuracy.
- Extensive experimental results show that MAEST outperforms state-of-the-art methods, achieving superior clustering performance.
- MAEST performs robustly across various sequencing platforms and data scales in ST, with the capability to seamlessly integrate multiple slices horizontally.

## Supplementary Material

Supplementary_material_bbaf086

## Data Availability

Datasets analyzed in this paper are available in raw form from their original authors (see Supplementary Tables S2).
