# Supplementary material for: MAEST: accurately spatial domain detection in spatial transcriptomics with graph masked autoencoder
Source: Brief Bioinform. 2025 Mar 7;26(2):bbaf086. doi: 10.1093/bib/bbaf086 (PMC11886571; doi:10.1093/bib/bbaf086)
Supplement: Supplementary_material_bbaf086 [file supplementary_material_bbaf086.pdf]

Supplementary Information

MAEST: Accurate Spatial Domain Detection in Spatial Transcriptomics with Graph Masked Autoencoder

Pengfei Zhu[1,2,†], Han Shu[1,2,†], Yongtian Wang[1,2,†] , Xiaofeng Wang[3], Yuan Zhao[1,2], Jialu Hu[1,2], Jiajie Peng[1,2], Xuequn Shang[1,2], Zhen Tian[4,*], Jing Chen[5,*] and Tao Wang[1,2,*]

[1] School of Computer Science, Northwestern Polytechnical University, 1 Dongxiang Rd., 710072, Xi'an, China,

[2] Key Laboratory of Big Data Storage and Management, Ministry of Industry and Information Technology, Northwestern Polytechnical University, 1 Dongxiang Rd., 710072, Xi'an, China,

[3] General Surgery Department, The Affiliated Hospital of Northwest University: Xi'an No 3 Hospital, 710018, Xi'an, China,

[4] School of Computer Science and Artificial Intelligence, Zhengzhou University, No.100 Science Avenue, 450001, Zhengzhou, China

[5] School of Computer Science and Engineering, Xi'an University of Technology, No.5 South Jinhua rd., 710048, Xi'an, China,

*Corresponding author. twang@nwpu.edu.cn, ieztian@zzu.edu.cn, or chen-jing@xaut.edu.cn

†These authors contributed equally to this work.

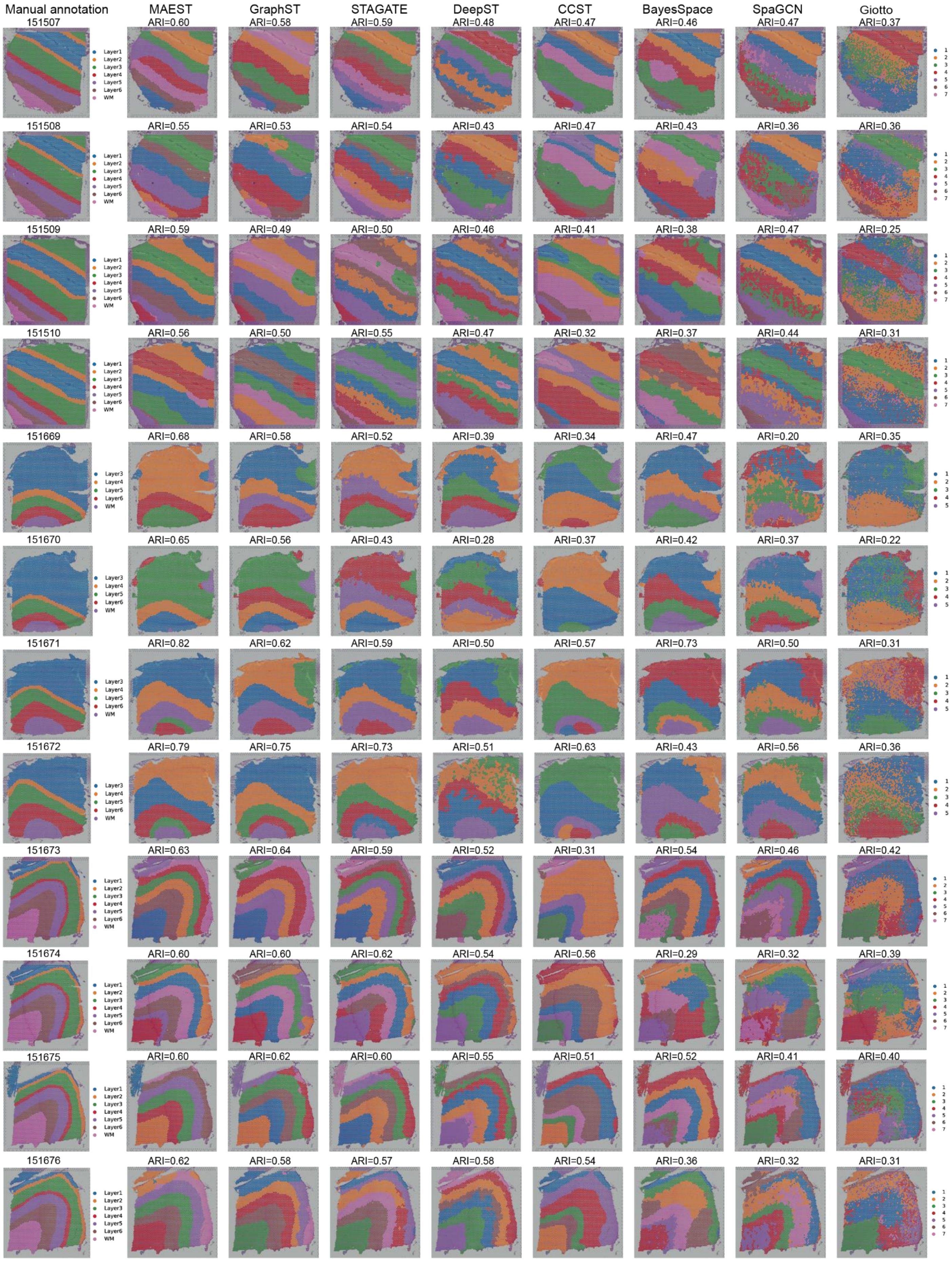

Fig. S1. Manual annotation and comparison of spatial domains by clustering assignments via MAEST, GraphST, STAGATE, DeepST, CCST, BayesSpace, SpaGCN and Giotto in all 12 sections of the DLPFC dataset.

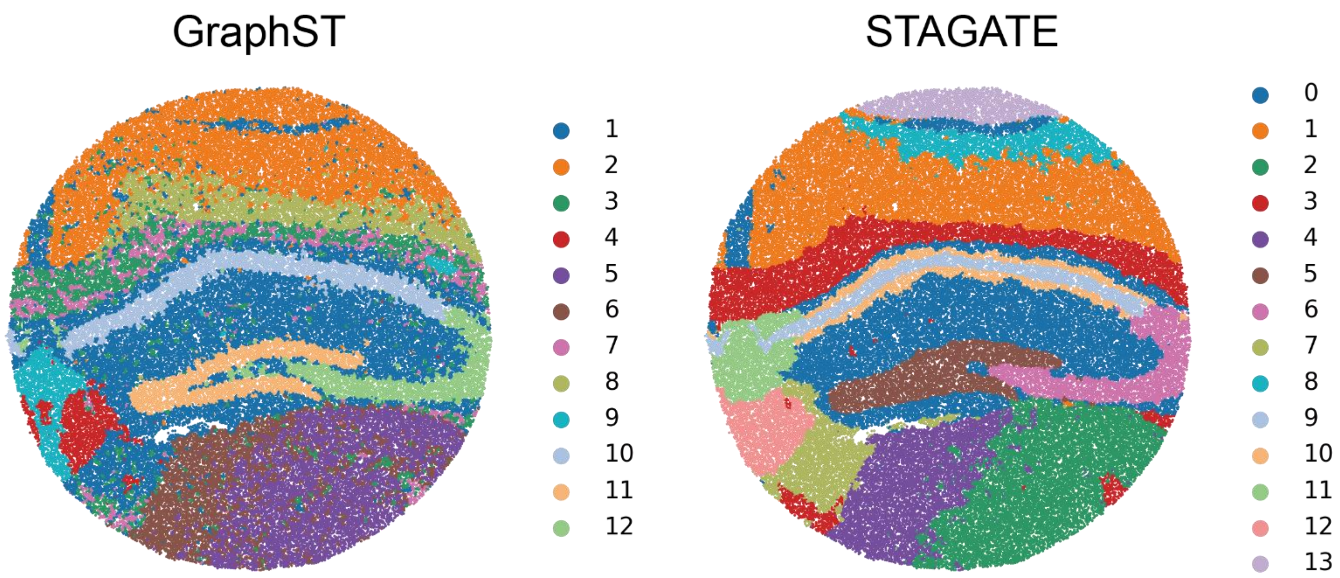

Fig. S2. Fine-grained clustering results of GraphST and STAGATE for mouse hippocampus dataset.

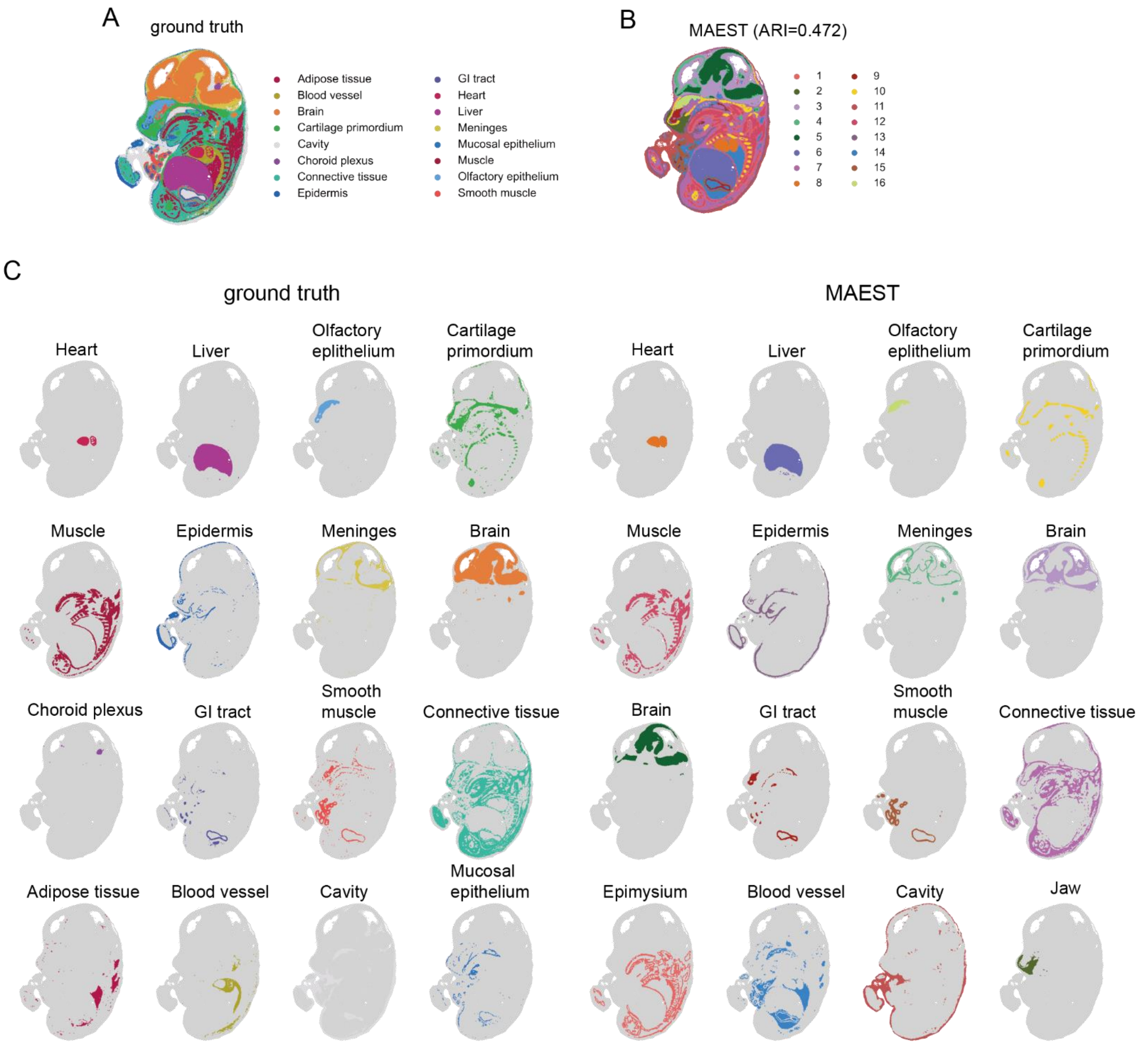

Fig. S3. Spatial clustering on the E14.5 mouse embryo Stereo-seq dataset. A. Ground truth annotations of the E14.5 stage mouse embryo dataset. **B.** Spatial clustering results of the E14.5 stage mouse embryo generated by MAEST. **C.** Visualization of spatial domains identified by the original Stereo-seq study and MAEST with 16 clusters.

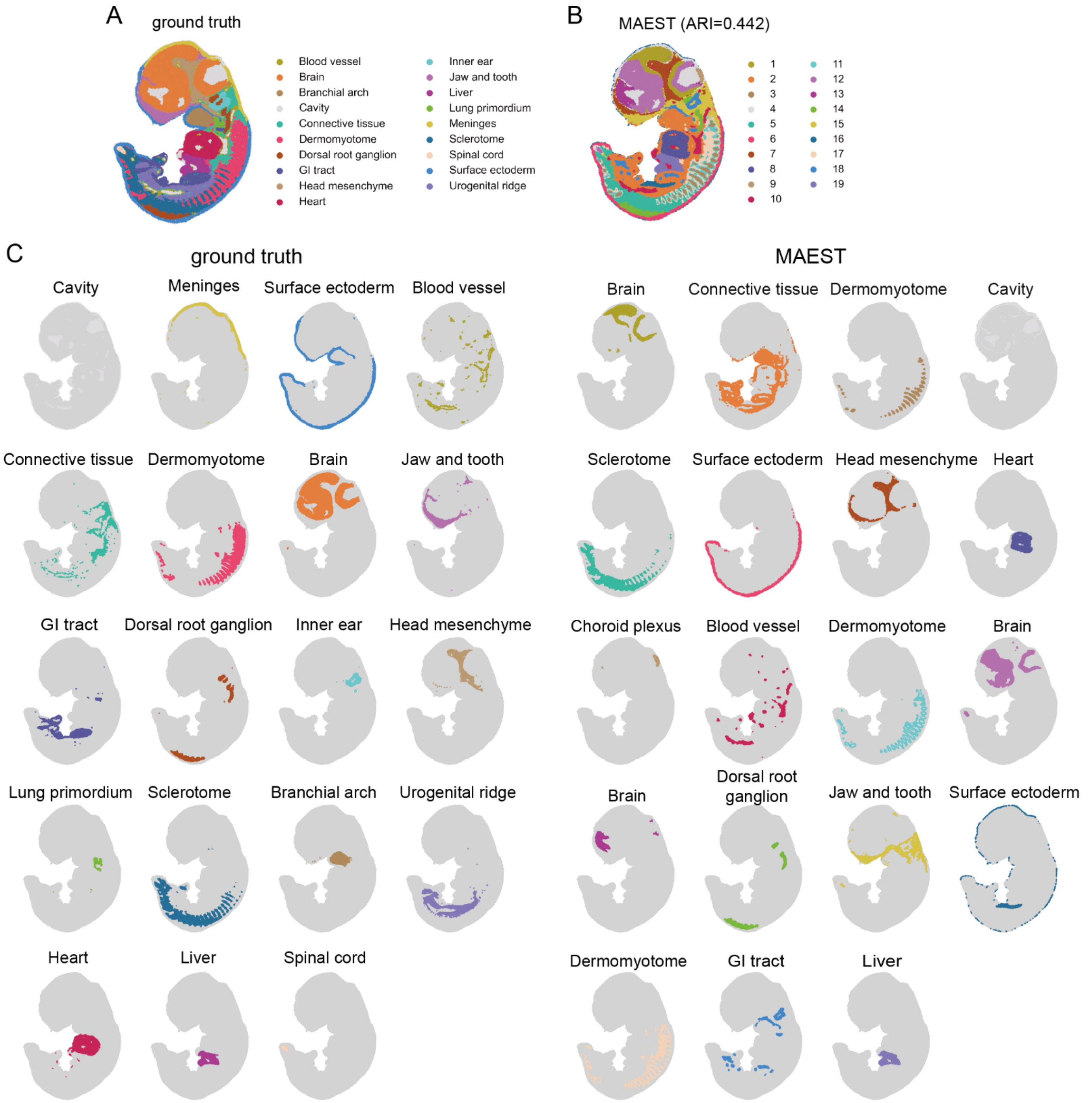

Fig. S4. Spatial clustering on the E11.5 mouse embryo Stereo-seq dataset. A. Ground truth annotations of the E11.5 stage mouse embryo dataset. **B.** Spatial clustering results of the E11.5 stage mouse embryo generated by MAEST. **C.** Visualization of spatial domains identified by the original Stereo-seq study and MAEST with 19 clusters.

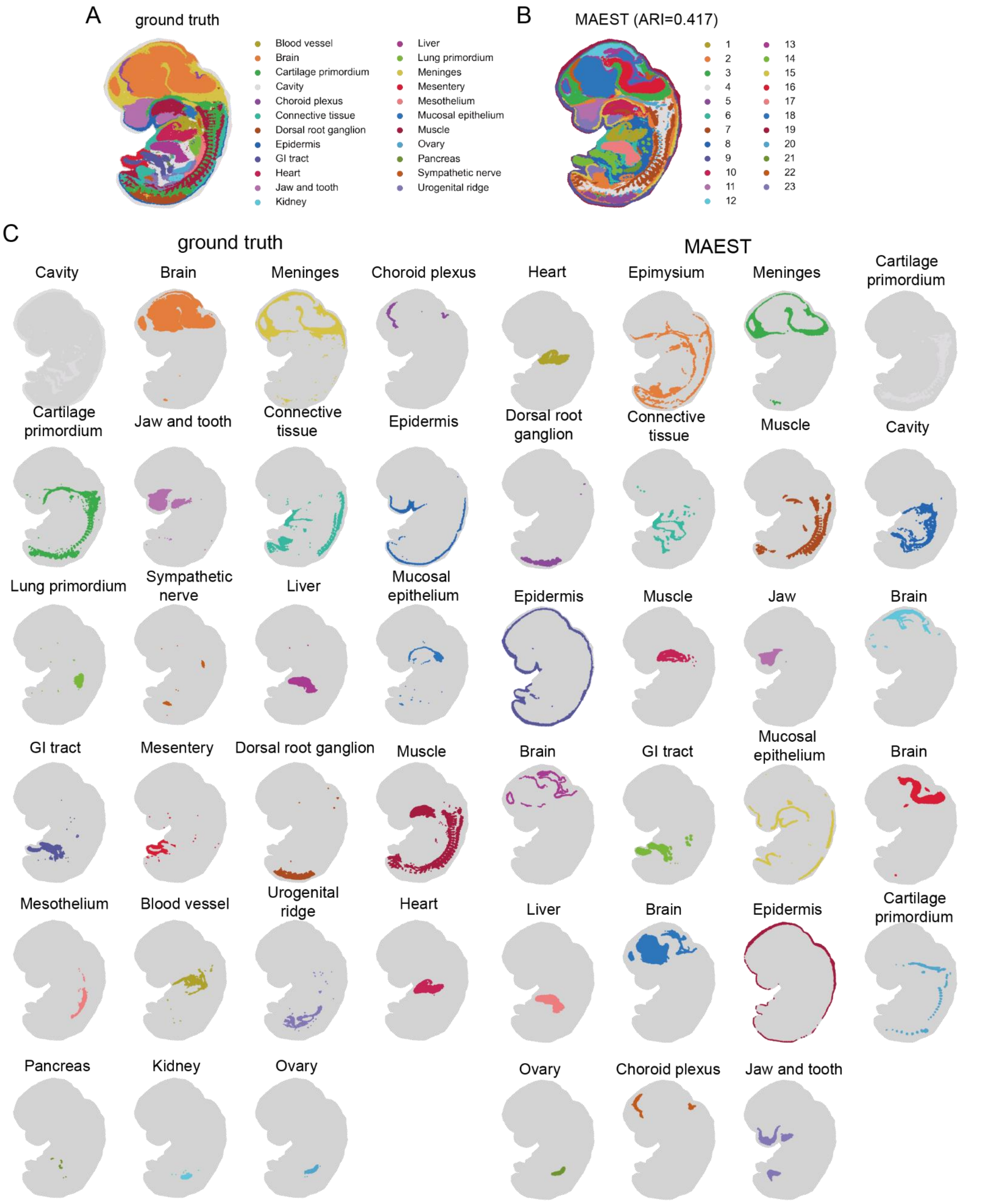

Fig. S5. Spatial clustering on the E12.5 mouse embryo Stereo-seq dataset. A. Ground truth annotations of the E12.5 stage mouse embryo dataset. **B.** Spatial clustering results of the E12.5 stage mouse embryo generated by MAEST. **C.** Visualization of spatial domains identified by the original Stereo-seq study and MAEST with 23 clusters.

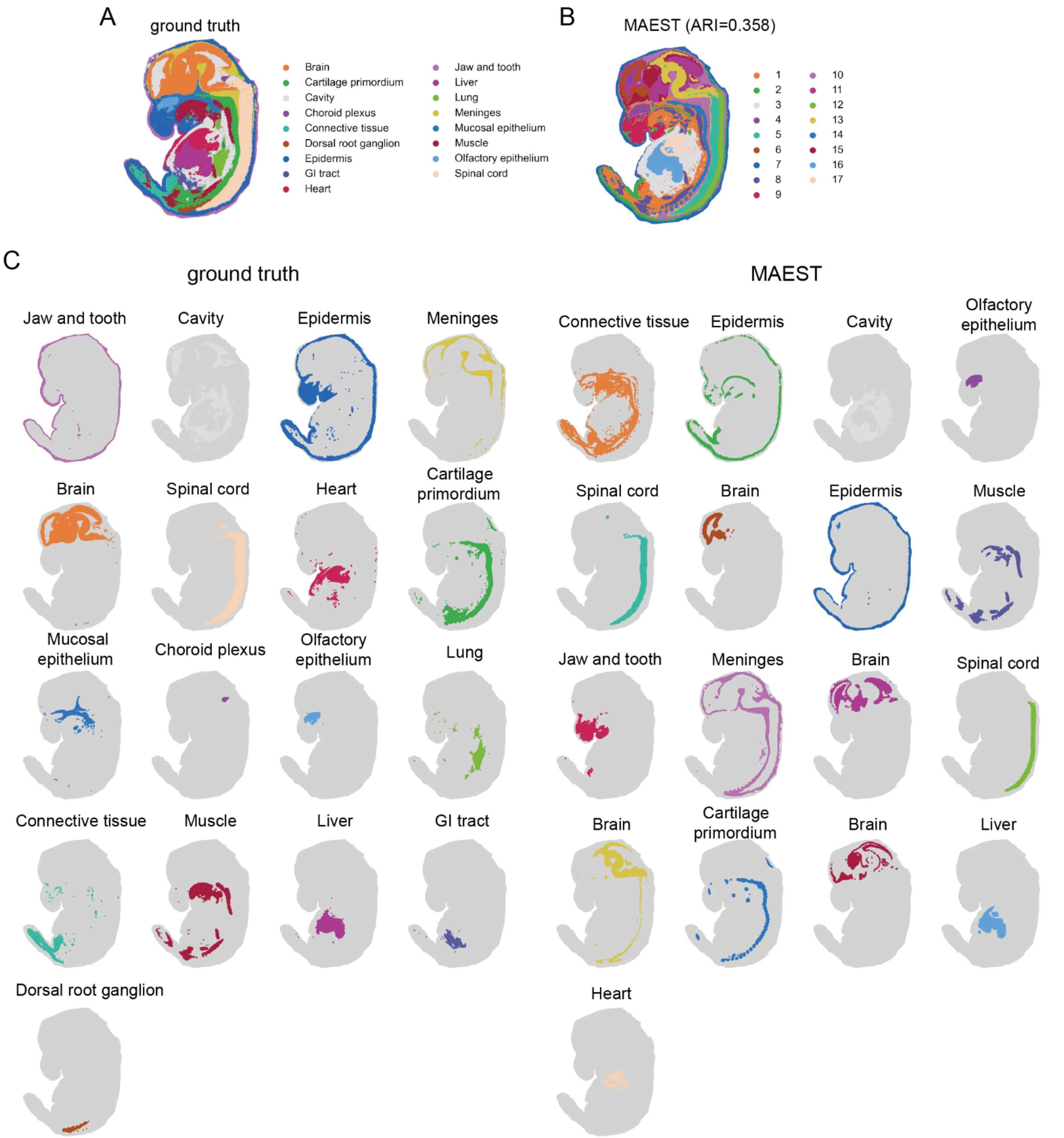

Fig. S6. Spatial clustering on the E13.5 mouse embryo Stereo-seq dataset. A. Ground truth annotations of the E13.5 stage mouse embryo dataset. **B.** Spatial clustering results of the E13.5 stage mouse embryo generated by MAEST. **C.** Visualization of spatial domains identified by the original Stereo-seq study and MAEST with 17 clusters.

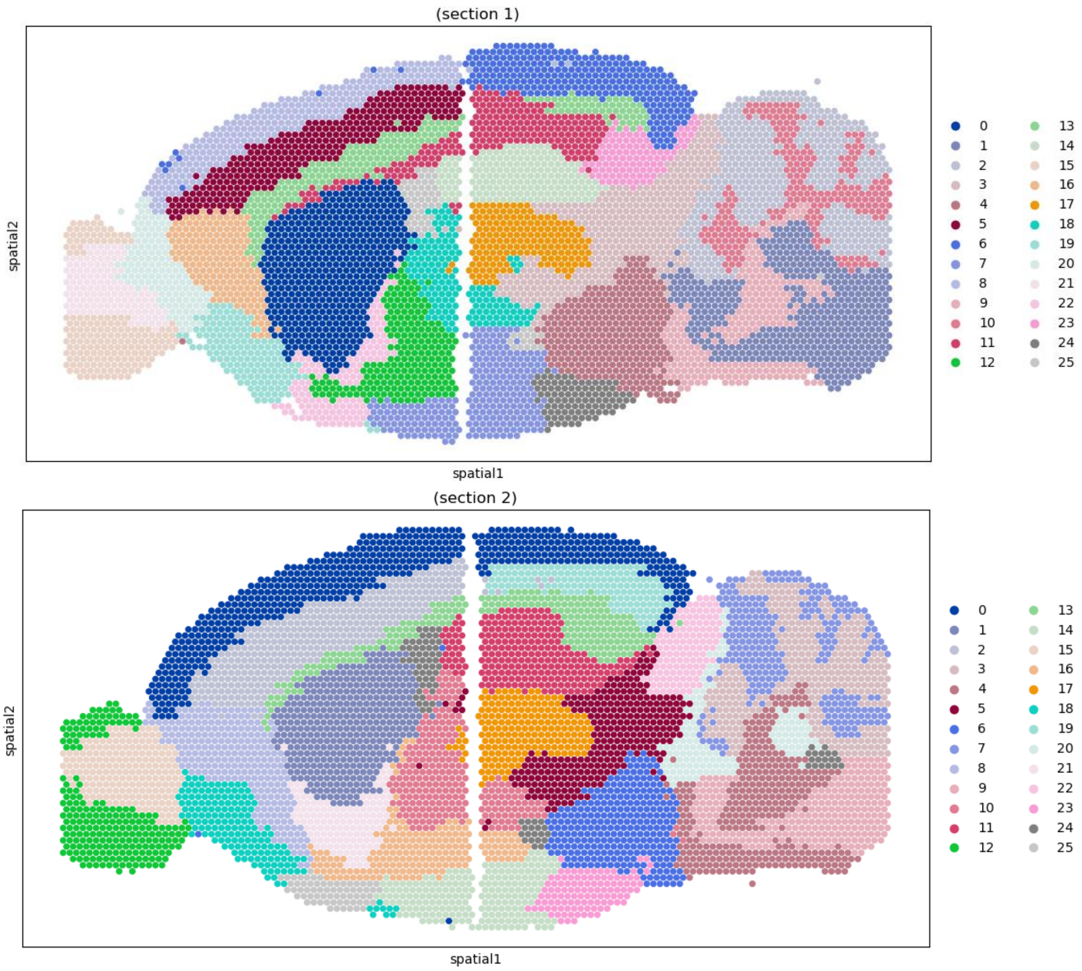

Fig. S7. The horizontal integration results of SpaGCN on two mouse brain sections, each comprising anterior and posterior slices.

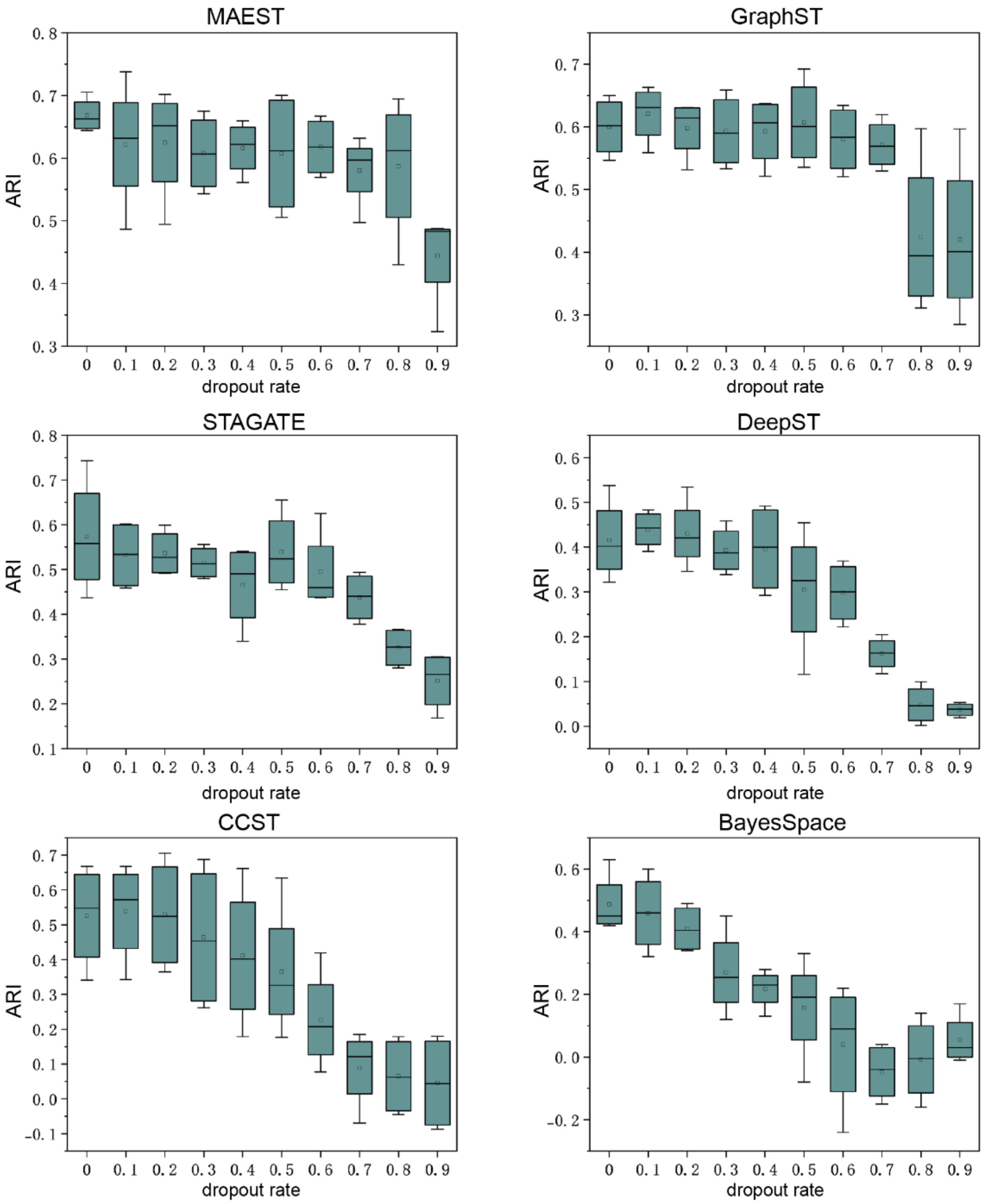

Fig. S8. The clustering performance of MAEST and five comparison methods varies with the dropout rate across four slices (151669–151672) of the DLPFC dataset.

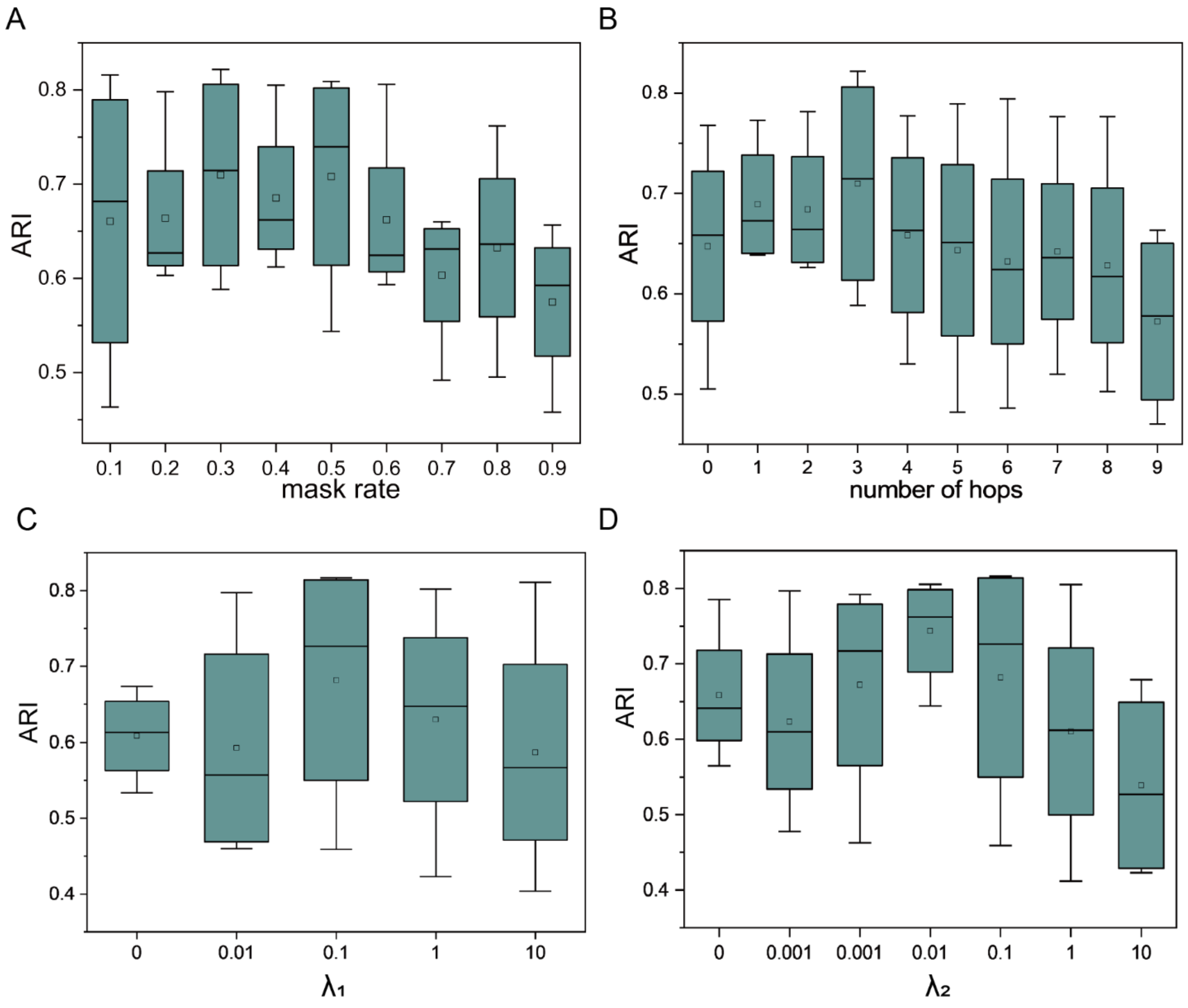

Fig. S9. The influence of hyperparameters on model performance. A. The impact of different masking rates on clustering results. **B.** The impact of different numbers of hops on clustering results. **C.** The Impact of different λ_1 weights on clustering results. **D.** The Impact of different λ_2 weights on clustering results.

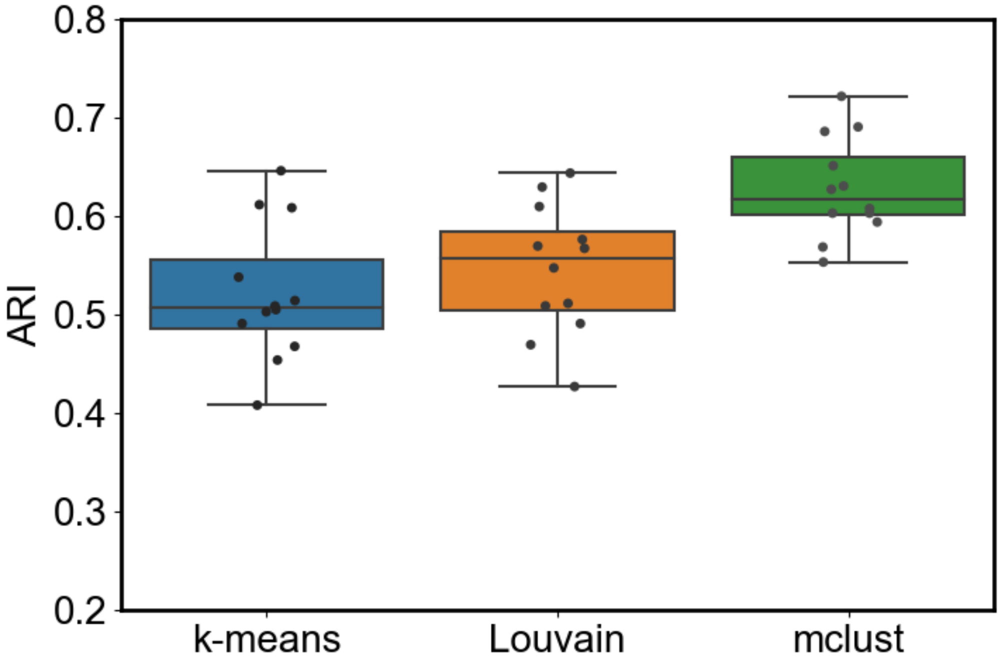

Fig. S10. Comparison analysis between k-means, Louvain and mclust with the output of MAEST as input with the DLPFC dataset.

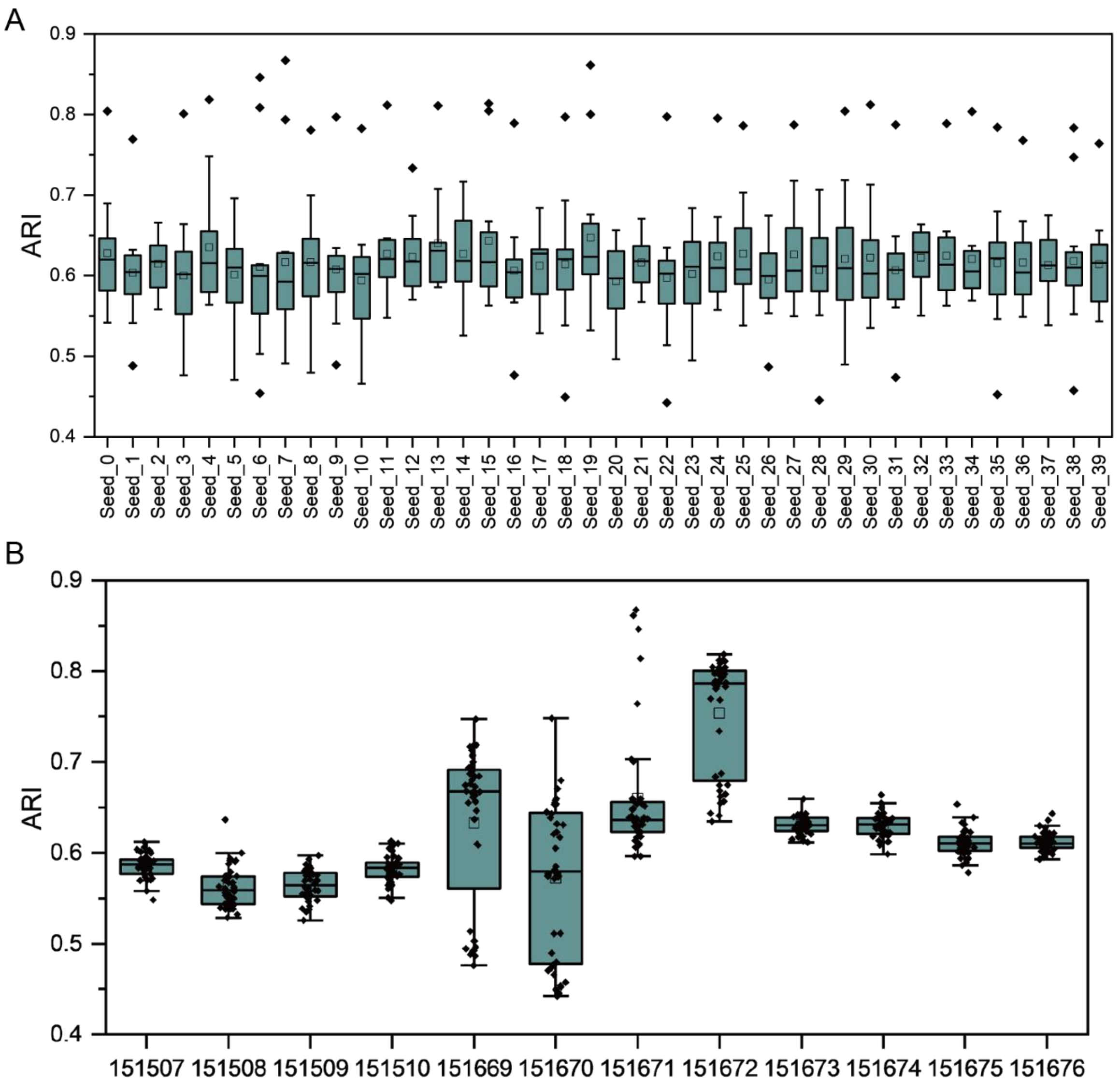

Fig. S11. The influence of random seeds on model robustness. A. The clustering accuracy of MAEST in all 12 sections under the default hyperparameters with different random seeds. **B.** The clustering accuracy for each section under the default hyperparameters with different random seeds (n=40 for each box).

Table S1. The state-of-the-art methods for embedding learning and spatial domain identification in spatial transcriptomics.

Method	Basic model	Use histology image	Source code link	Reference
Giotto	HMRF	No	https://github.com/drieslab/Giotto	Dries et al.,Genome biology,2021[1]
BayesSpace	Bayesian	No	https://github.com/edward130603/BayesSpace	zhao et al., Nat. Biotechnol., 2021[2]
SpaGCN	GCN	Yes	https://github.com/JinmiaoChenLab/SEDR	Hu et al., Nat. Methods, 2021[3]
DeepST	GCN,VAE	Yes	https://github.com/JiangBioLab/DeepST	Xu et al., Nucleic Acids Res., 2022[4]
CCST	DGI	No	https://github.com/xiaoyeye/CCST	Li et al., Nat. Comput. Sci., 2022[5]
STAGATE	GAT,AE	No	https://github.com/QIFEIDKN/STAGATE_pyG	Dong and Zhang, Nat. Commun., 2022[6]
GraphST	GCN,DGI,AE	No	https://github.com/JinmiaoChenLab/GraphST	Long et al., Nat. Commun., 2023[7]

Table S2. Summary of all datasets used in this study.

Platform	Tissue	Section	#Spots/Bins	Related figures	Reference
10x Visium	Human dorsolateral prefrontal cortex (DLPFC)	151507	4,226	Fig.2A-B, Fig.S1, Fig.S4C	[8]
		151508	4,384	Fig.2A, Fig.S1, Fig.S4C	
		151509	4,789	Fig.2A, Fig.S1, Fig.S4C	
		151510	4,634	Fig.2A, Fig.S1, Fig.S4C	
		151669	3,661	Fig.2A, Fig.S1, Fig.S4A-C	
		151670	3,498	Fig.2A, Fig.S1, Fig.S4A-C	
		151671	4,110	Fig.2A-C, Fig.S1, Fig.S4A-C	
		151672	4,015	Fig.2A, Fig.S1, Fig.S4A-C	
		151673	3,639	Fig.2A-B, Fig.S1, Fig.S4C	
		151674	3,673	Fig.2A, Fig.S1, Fig.S4C	
		151675	3,592	Fig.2A, Fig.S1, Fig.S4C	
		151676	3,460	Fig.2A, Fig.S1, Fig.S4C	
	Mouse brain	Mouse Brain Section 1 (Sagittal-Anterior)	2,695	Fig.5.C-E	[3]
		Mouse Brain Section 1 (Sagittal-Posterior)	3,355	Fig.5.C-E	
		Mouse Brain Section 2 (Sagittal-Anterior)	2,825	Fig.5.C-E	
		Mouse Brain Section 2 (Sagittal-Posterior)	3,289	Fig.5.C-E	
Stereo-seq	Mouse olfactory bulb	Puck_200127_15	19,109	Fig.3G-H	[9]
	Mouse embryo	E11.5	30,124	Fig.4E	
		E12.5	51,365	Fig.4E	
		E13.5	73,168	Fig.4E	
		E14.5	92,928	Fig.4A-E, Fig.S3A-C	
Slide-seqV2	Mouse hippocampus	Puck_200115_08	52,869	Fig.3B-E, Fig.S2	[10]

Table S3. Clustering performance (%) of MAEST and seven state-of-the-art baselines on 12 slices of the DLPFC dataset.

Method	Metric	151507	151508	151509	151510	151669	151670	151671	151672	151673	151674	151675	151676	mean
Giotto	ACC	54.49	59.26	44.63	51.8	54.18	45.81	47.52	56.58	59.07	58.76	57.23	47.48	53.07
	ARI	37.35	36.87	25.11	31.73	35.19	22.73	31.04	36.11	42.17	39.88	40.24	31.8	34.19
	NMI	46.81	45.7	38.48	41.99	41.87	36.36	43.64	40.78	49.69	45.85	45.78	41.95	43.24
SpaGCN	ACC	62.69	48.3	65.54	62.48	46.92	63.35	66.16	69.6	57.38	50.12	55.69	48.88	58.09
	ARI	47.13	36.03	47.92	44.93	20.14	37.41	50.41	56.55	46.32	32.38	41.28	32.69	41.10
	NMI	59.04	45.84	60.43	57.04	35.61	48.24	61.53	65.54	62.73	48.67	52.36	51.9	54.08
BayesSpace	ACC	61.08	57.73	60.84	55.82	66.86	64.44	80.89	55.17	68.73	46.33	61.95	52.11	61.00
	ARI	46.9	43.69	38.14	37.67	47.04	42.91	73.34	43.89	54.99	29.59	52.97	36.75	45.66
	NMI	62.79	59.91	59.24	55.3	60.99	55.46	68.89	59.63	68.8	48.18	68.47	56.66	60.36
CCST	ACC	55.34	62.57	54.97	49.4	50.44	67.05	68.39	65.61	55.61	70.18	67.61	69.02	61.35
	ARI	47.52	47.56	41.03	32.67	34.09	37.27	67.9	63.44	31.27	56.98	51.98	54.68	47.20
	NMI	67.59	60.89	62.32	57.1	54.43	48.09	66.29	66.14	53.99	67.46	64.53	65.86	61.22
DeepST	ACC	64.91	56.11	59.29	64.27	57.73	48.45	63.82	64.27	68.87	68.97	65.4	**77.09**	63.27
	ARI	48.51	43.51	46.97	47.94	39.35	28.26	50.26	51.23	52.12	54.33	55.44	58.36	48.02
	NMI	63.25	54.37	62.26	61.57	61.78	49.07	64.62	64.45	66.18	67.47	66.37	70.02	62.62
STAGATE	ACC	77.99	**71.99**	69.65	**74.21**	71.34	65.56	74.54	80.79	76.24	71.75	73.64	72.31	73.33
	ARI	59.88	54.26	50.79	55.13	52.13	43.68	59.6	73.98	59.26	**62.82**	**60.93**	57.06	57.46
	NMI	70.27	**67.35**	67.01	**67.18**	63.32	57.4	69.62	72.39	71.67	74	**71.19**	67.92	68.28
GraphST	ACC	71.62	65.94	66.96	69.12	74.31	75.72	76.28	81.58	**78.7**	71.58	**76.05**	71.93	73.32
	ARI	58.06	53.62	49.72	50.33	58.15	56.23	62.84	75.01	**64.62**	60.96	62.36	58	59.16
	NMI	70.5	61.33	67.1	64.53	64.54	60.62	72.83	72.7	**74.03**	70.9	70.54	67.23	68.07
MAEST	ACC	**78.58**	68.02	**74.87**	70.92	**77.59**	**79.45**	**84.44**	**82.74**	78.01	**71.64**	74.06	75.69	**76.33**
	ARI	**60.3**	**55.33**	**59.4**	**56.86**	**68.6**	**65.12**	**82.17**	**79.06**	63.06	60.3	60.77	**62.72**	**64.47**
	NMI	**71.12**	66.9	**70.28**	64	**67.07**	**63.54**	**77.11**	**75.84**	73.36	**73.08**	71.12	**70.61**	**70.34**

(Bold numbers represent the best performance for each slice.)

Reference

1. Dries, R. *et al.* Giotto: a toolbox for integrative analysis and visualization of spatial expression data. *Genome Biol* 22, 78 (2021).
2. Zhao, E. *et al.* BayesSpace enables the robust characterization of spatial gene expression architecture in tissue sections at increased resolution. Preprint at https://doi.org/10.1101/2020.09.04.283812 (2020).
3. Hu, J. *et al.* SpaGCN: Integrating gene expression, spatial location and histology to identify spatial domains and spatially variable genes by graph convolutional network. *Nat Methods* 18, 1342–1351 (2021).
4. Xu, C. *et al.* DeepST: identifying spatial domains in spatial transcriptomics by deep learning. *Nucleic Acids Research* 50, e131–e131 (2022).
5. Li, J., Chen, S., Pan, X., Yuan, Y. & Shen, H. *CCST: Cell Clustering for Spatial Transcriptomics Data with Graph Neural Network*. https://www.researchsquare.com/article/rs-990495/v1 (2021) doi:10.21203/rs.3.rs-990495/v1.
6. Dong, K. & Zhang, S. Deciphering spatial domains from spatially resolved transcriptomics with an adaptive graph attention auto-encoder. *Nat Commun* 13, 1739 (2022).
7. Long, Y. *et al.* Spatially informed clustering, integration, and deconvolution of spatial transcriptomics with GraphST. *Nat Commun* 14, 1155 (2023).
8. Maynard, K. R. et al. Transcriptome-scale spatial gene expression in the human dorsolateral prefrontal cortex. Nat. Neurosci. 24, 425–436 (2021).
9. Chen, A. et al. Spatiotemporal transcriptomic atlas of mouse organogenesis using DNA nanoball-patterned arrays. Cell 185, 1777-1792.e21 (2022).
10. https://singlecell.broadinstitute.org/single_cell/study/SCP354/slide-seq-study.
